# Effects of Continuous Care on Clinical Outcomes, Psychosocial Well‐Being, and Healthcare Utilization in Adults With Enterostomy: A Systematic Review and Meta‐Analysis of Randomized Controlled Trials

**DOI:** 10.1155/jonm/1161340

**Published:** 2026-10-19

**Authors:** Ru Yang, Zeyao Shi, Yingxin Li, Xi Huang, Xia Li, Xushu Chen, Qiong Chen, Yanling Hu, Yuan Li, Xiaowen Li

**Affiliations:** ^1^ Department of Neonatology West China Second University Hospital Sichuan University Chengdu China scu.edu.cn; ^2^ Key Laboratory of Birth Defects and Related Diseases of Women and Children Sichuan University Ministry of Education Chengdu China meb.gov.tr; ^3^ Department of Nursing West China Second University Hospital Sichuan University Chengdu China scu.edu.cn

**Keywords:** continuous care, enterostomy, healthcare utilization, quality of life, stoma-related complications, systematic review

## Abstract

**Aim:**

To evaluate the effects of continuous care on clinical outcomes, psychosocial well‐being, and healthcare utilization in adults with enterostomy.

**Background:**

Adults with enterostomy confront significant physical, psychological, and economic burdens. Continuous care has emerged as a promising intervention facilitating the critical hospital‐to‐home transition, yet its comprehensive effectiveness requires systematic evaluation.

**Evaluation:**

MEDLINE, Embase, CENTRAL, and CINAHL were searched from January 2000 to September 2026. Eligible studies were randomized controlled trials evaluating continuous care interventions in adults with enterostomy. The primary outcome was the incidence of stoma‐related complications; secondary outcomes included psychosocial well‐being (quality of life, self‐care ability, self‐efficacy, ostomy adjustment, anxiety, depression, and patient satisfaction) and healthcare utilization. Two researchers independently screened literature, extracted data, and assessed risk of bias using RoB 2. Random‐effects meta‐analyses were conducted in *R*, and certainty of evidence was assessed using GRADE.

**Key Issue(s):**

Eighteen trials involving 1926 participants were included. Continuous care significantly reduced stoma‐related complications (RR = 0.34; 95% CI: 0.26–0.45; *I* ^2^ = 0%) and improved quality of life (SMD = 1.55; 95% CI: 0.91–2.19; *I* ^2^ = 93.1%), self‐care ability (SMD = 1.57; 95% CI: 0.82–2.32; *I* ^2^ = 95.3%), and self‐efficacy (SMD = 1.29; 95% CI: 0.64–1.94; *I* ^2^ = 91.9%). Beneficial effects were also observed for ostomy adjustment, anxiety, and depression. Narrative synthesis of three studies suggested fewer unplanned readmissions.

**Conclusions:**

Continuous care interventions may improve clinical and psychosocial outcomes and reduce unplanned readmissions in adults with enterostomy. However, because several patient‐reported outcomes were supported by low or very low certainty evidence and showed substantial heterogeneity, these findings should be interpreted as supportive rather than definitive evidence for early, seamless continuous care models.

**Implications for Nursing Management:**

Nurse managers should consider developing structured hospital‐to‐home pathways incorporating discharge preparation, self‐care readiness assessment, planned follow‐up, and access to specialist or multidisciplinary support.

## 1. Introduction

Enterostomy formation, encompassing both colostomy and ileostomy, represents a pivotal life‐saving surgical intervention performed globally. In the United States alone, approximately 100,000 patients undergo this procedure annually [1, 2], with an estimated 725,000–1,000,000 individuals currently living with an ostomy [3]. This procedure is indicated for a broad spectrum of pathologies, ranging from colorectal cancer to benign conditions such as inflammatory bowel disease, diverticulitis, and trauma [4, 5]. Regardless of the etiology, the creation of a stoma necessitates a fundamental alteration in physiological function, diverting the fecal stream and requiring patients to adapt to a new mode of excretion that profoundly reshapes their daily lives [6, 7].

Living with an enterostomy imposes significant physical, psychological, and social burdens. A primary concern in postoperative care is the maintenance of tissue integrity; stoma‐related complications are prevalent, with peristomal skin complications affecting approximately 36%–78% of individuals [8, 9]. The corrosive nature of effluent often compromises the peristomal skin barrier, leading to irritant contact dermatitis that significantly impedes quality of life (QoL) and increases the cost of treatment [10–12]. Beyond physical sequelae, the visible alteration of body image precipitates substantial psychosocial distress [13]. Systematic reviews have reported pooled prevalence rates of 47.6% for anxiety and 38.9% for depression among ostomy patients [14, 15], levels substantially exceeding those in the general population.

The transition from the hospital to the home environment constitutes a period of heightened vulnerability. With the widespread adoption of enhanced recovery after surgery (ERAS) protocols, hospital length of stay has been notably reduced [16–18], frequently resulting in patients being discharged before achieving proficiency in self‐management skills. This premature cessation of professional supervision often leaves patients ill‐equipped to manage stoma appliances or recognize early signs of tissue damage [19, 20]. Consequently, healthcare utilization remains alarmingly high; recent evidence from a large US database indicates that hospital readmission rates can reach 42% within 120 days post‐discharge [10], with ostomy‐related complications accounting for a significant proportion of these events. Such patterns impose a substantial economic burden on both patients and healthcare systems.

To bridge the gap between hospital discharge and effective home adaptation, continuous care has emerged as a critical component of comprehensive stoma management. In this review, continuous care was defined as a coordinated, planned, and patient‐centered transitional‐care intervention designed to maintain professional support across the hospital‐to‐home transition and to promote home‐based self‐management and long‐term adaptation [21]. Across eligible studies, the core active components commonly included transition preparation or discharge planning, structured stoma‐care education and skills training, scheduled professional follow‐up, individualized problem solving, psychosocial support, and timely access to specialist advice. These components could be delivered in person or remotely and were often led by specialized or trained nurses [22]. The intended mechanisms were to enhance stoma adaptability, strengthen self‐care ability and self‐efficacy, support psychosocial adjustment, reduce complications, and prevent avoidable readmissions [23–26].

Although previous studies and systematic reviews have examined continuing care, education, telehealth, self‐care, or adjustment in people with stomas, important evidence gaps remain [22, 27–30]. Existing reviews often combine broad nursing or educational interventions, include mixed enterostomy and urostomy populations, examine a single outcome domain, or incorporate nonrandomized designs. These differences limit conclusions specifically about structured professional support across the hospital‐to‐home transition. Previous syntheses have also not consistently distinguished intervention typologies and delivery personnel or assessed risk of bias separately for objective and patient‐reported outcomes and the certainty of evidence across clinical, psychosocial, and healthcare utilization outcomes. Therefore, this systematic review and meta‐analysis identified, appraised, and synthesized RCTs evaluating continuous care compared with usual care in adults with enterostomy. Specifically, the review addressed the following questions:

• What is the impact of continuous care on clinical outcomes (i.e., incidence of stoma‐related complications) in adult patients with enterostomy?
• How does continuous care affect psychosocial well‐being, including QoL, self‐care ability, self‐efficacy, ostomy adjustment, anxiety, depression, and patient satisfaction?
• What is the effect of continuous care on healthcare utilization, such as hospital readmission rates, length of hospital stays, and healthcare costs in this population?

## 2. Methods

### 2.1. Study Registration and Design

This systematic review and meta‐analysis was prospectively registered in PROSPERO (CRD42024595335). The review methodology followed the rigorous guidelines outlined in the Cochrane Handbook for Systematic Reviews of Interventions [31] and was reported in compliance with the Preferred Reporting Items for Systematic Reviews and Meta‐Analyses (PRISMA) 2020 Statement (Supporting information 1) [32].

### 2.2. Data Sources and Search Strategy

A systematic literature search was conducted in MEDLINE (via OVID), Embase (via OVID), CINAHL (via EBSCOhost), and the Cochrane Central Register of Controlled Trials (CENTRAL via Cochrane Library). The search spanned from January 2000 to September 2024, with a final update conducted in September 1, 2026. No language restrictions were applied during searching or study selection. The search strategy was constructed in consultation with a medical librarian using Medical Subject Headings (MeSH) and free‐text terms for enterostomy, continuity or transition of care, discharge planning, home care, telemedicine or mobile health, and randomized trials. Database‐specific strategies are provided in Supporting information 2. Additionally, bibliographic hand‐searching was performed on the reference lists of all included studies and relevant reviews to identify further eligible studies. Clinical trial registries (e.g., World Health Organization International Clinical Trial Registry Platform) were also scrutinized for potential unpublished or ongoing studies.

### 2.3. Inclusion and Exclusion Criteria

#### 2.3.1. Studies Were Selected Based on the PICOS Framework

• *Population:* Adult patients (aged ≥ 18 years) who underwent temporary or permanent enterostomy (ileostomy or colostomy).
• *Intervention:* Continuous care interventions designed to facilitate the hospital‐to‐home transition and provide sustained support. Eligible interventions were required to include a structured transitional‐support element, such as planned education or discharge preparation, self‐management skills training, scheduled follow‐up, ongoing professional guidance, remote monitoring, or individualized problem solving. These interventions could be initiated at any point from preadmission through the immediately postdischarge period (within 1 week), with support directed toward the postdischarge adaptation phase. This temporal window was selected to capture the early transition phase, address acute postdischarge challenges, reinforce self‐management skills, and enable timely identification of complications, thereby reflecting the core tenet of seamless continuous care.
• *Comparison:* Usual care, routine care, or standard care as defined by the original studies, but did not include a structured, proactive, and longitudinal continuous care program.
• *Outcomes:* Studies were required to report at least one of the following domains:
   Primary outcome: Clinical outcomes, specifically defined as the incidence of stoma‐related complications.
   Secondary outcomes: (1) Psychosocial well‐being: QoL, self‐care ability, self‐efficacy, ostomy adjustment, anxiety, depression, and patient satisfaction. (2) Healthcare utilization: Hospital readmission rates, length of hospital stays, and healthcare costs.
• *Study design:* Only RCTs.

Studies were excluded if they (1) involved pediatric patients or lacked separate adult enterostomy data; or (2) included mixed stoma types without separate enterostomy data.

### 2.4. Study Selection and Data Extraction

The retrieved articles were imported into EndNote for deduplication. Two investigators independently screened titles and abstracts according to predefined inclusion and exclusion criteria. Following this initial screening, full‐text articles of potentially eligible studies were retrieved and reviewed independently by the same investigators to confirm final inclusion. Any disagreements at either stage were resolved through discussion or consultation with a third reviewer. A PRISMA flow diagram was used to document the selection process.

The same two reviewers independently extracted data using a prepiloted data extraction form. Extracted information included (1) study characteristics (e.g., first author, publication year, country); (2) study design (e.g., design, setting, sample size); (3) participant characteristics (e.g., age, gender, diagnosis, stoma type, stoma status); (4) intervention details (e.g., intervention category, content, delivery personnel, initiation of intervention, duration); (5) control group details; and (6) outcome measures (e.g., measurement tools, time points of assessment). Any discrepancies were resolved by consensus, with arbitration from a third reviewer when necessary.

### 2.5. Risk‐of‐Bias Assessment

Two reviewers independently assessed risk of bias of the included RCTs using the revised Cochrane risk‐of‐bias tool (ROB 2) [33]. This tool evaluates five domains: (1) bias arising from the randomization process, (2) bias due to deviations from intended interventions, (3) bias due to missing outcome data, (4) bias in outcome measurement, and (5) bias in selection of the reported result. Given the differing susceptibilities to bias, patient‐reported subjective outcomes (e.g., QoL) and objective outcomes (e.g., complications) were assessed separately where appropriate. Each study was classified as having “low risk,” “some concerns,” or “high risk” of bias according to the RoB 2 framework. Discrepancies between reviewers were resolved through discussion or consultation with a third reviewer. The results of the risk‐of‐bias assessments were presented graphically. The certainty of evidence for key outcomes was assessed using GRADE and categorized as “high,” “moderate,” “low,” or “very low” [34].

### 2.6. Data Analysis

A narrative synthesis was conducted for all included studies to summarize study characteristics, interventions, and populations. When studies were deemed sufficiently homogeneous, meta‐analyses were performed using *R* software (version 4.3.2). A random‐effects model was applied for all pooled analyses to account for anticipated clinical and methodological heterogeneity.

For continuous outcomes, the standardized mean difference (SMD) with 95% confidence intervals (CIs) was calculated when different measurement scales were used; otherwise, the mean difference (MD) with 95% CIs was employed. For dichotomous outcomes, risk ratios (RRs) with 95% CIs were calculated. Heterogeneity was assessed via visual inspection of forest plots and quantified using the *I* ^2^ statistic and Cochran’s *Q* test. *I* ^2^ values of 0%–40%, 30%–60%, 50%–90%, and 75%–100% were considered unimportant, moderate, substantial, and considerable heterogeneity, respectively [31]. Exploratory subgroup analyses for stoma‐related complications examined intervention typology and delivery personnel. Sensitivity analyses sequentially omitted each study and excluded studies at high risk of bias. Publication bias was evaluated using funnel plots and Egger’s test [35] contingent upon the availability of ≥ 10 studies for a specific outcome.

### 2.7. Protocol Deviations

The registered protocol prespecified exploratory analyses by patient and intervention characteristics and proposed meta‐regression when sufficient studies were available. However, it did not specify the two intervention typologies used in this review or limit subgroup analyses to the primary outcome. During synthesis, the typology labels were developed from the prespecified intervention features to organize clinically diverse continuous care models. Because the secondary meta‐analyses included only 3–9 studies, with sparse and uneven distributions across candidate covariates, subgroup or moderator analyses for secondary outcomes and meta‐regression were not conducted; subgroup analyses were therefore limited to stoma‐related complications.

## 3. Results

### 3.1. Search Results

A total of 6337 records were retrieved from the four databases. After removing 1646 duplicates, 4691 records remained for title and abstract screening, of which 4556 were excluded for not meeting the inclusion criteria. Subsequently, 123 full‐text articles were assessed for eligibility, and 108 were excluded. An additional three studies were identified through reference list screening. Eighteen studies [23, 24, 26, 36–50] were therefore included in the final analysis. The study selection process is illustrated in the PRISMA flow diagram (Figure 1). Reasons for excluding the 105 studies at the full‐text review stage are provided in Supporting Table 1.

**FIGURE 1:**
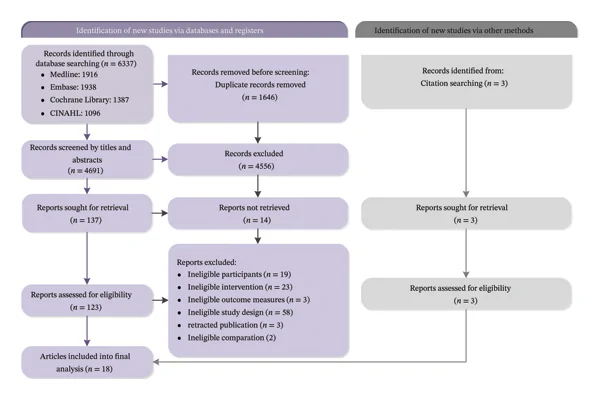
The PRISMA flow diagram of the study selection.

### 3.2. Study Characteristics

The characteristics of the included studies are summarized in Table 1 and further detailed in Supporting Table 2. The 18 included RCTs, published between 2013 and 2026, were predominantly conducted in China (*n* = 15) [23, 24, 26, 36–41, 43, 45–49], with three studies conducted in Turkey [42, 44, 50]. The aggregate sample comprised 1926 participants (intervention: *n* = 963; control: *n* = 963), with individual study sample sizes ranging from 48 [40] to 200 [49]. Across studies, the mean age of participants ranged from 43.7 (SD 4.1) [48] to 62.7 (SD 10.2) years [36] in the intervention groups and from 44.6 (SD 4.3) [48] to 64.2 (SD 10.2) years [44] in the control groups. Female participation rates varied from 25.0% [44] to 53.3% [42], with colorectal cancer being the primary diagnosis for the majority of individuals.

**TABLE 1:** Characteristics of the included studies (*n* = 18).

Study (country)	Participants (I vs. C)	Intervention group (I vs. C)	Control group	Outcomes
Geng et al. [47] (China)	N: 60 (30/30)	Category: Technology‐mediated postdischarge support	Routine care	• Complications
	Age: 45.4/45.4	Provider: Nurse‐led		• QoL (Not specified)
	Female: 41.7%	Duration: 6 months		• SAS and SDS
	Type: Mixed^a^	Core components: WeChat group for real‐time consultation, weekly telephone follow‐ups, family stoma care training, and organized social activities.		• Satisfaction
	Status: Not specified			

Hu et al. [45] (China)	N: 108 (54/54)	Category: Technology‐mediated postdischarge support	Routine care	• Complications
	Age: 54.2/53.8	Provider: MDT		• SF‐36
	Female: 45.4%	Duration: 3 months		• ESCA
	Type: Colostomy	Core components: MDT provided online consultation, pushed health education articles, and answered questions via WeChat.		• SAS and SDS
	Status: Temporary			• Satisfaction

Li 2024 [41] (China)	N: 82 (41/41)	Category: Comprehensive perioperative support	Routine care	• Stoma‐QoL
	Age: 62.5/55.5	Provider: Nurse‐led (Orem’s theory)		• Ostomy self‐care ability scale
	Female: 50.0%	Duration: 3 months		• Readmission and LOS
	Type: Mixed^a^	Core components: Stoma care training during hospitalization, discharge readiness assessment, and postdischarge telephone support.		
	Status: Mixed^b^			

Lin et al. [24] (China)	N: 153 (78/75)	Category: Comprehensive perioperative support	Routine care	• Complications
	Age: 61.7/60.4	Provider: MDT		• Stoma‐QoL
	Female: 30.7%	Duration: 3 months		• SSES
	Type: Mixed^a^	Core components: Needs assessment, MDT education (videos, manuals), discharge teaching/readiness, and regular WeChat/phone follow‐ups.		• Readmission and LOS
	Status: Not specified			

Lin et al. [46] (China)	N: 124 (62/62)	Category: Comprehensive perioperative support	Routine care	• Complications
	Age: 62.7/61.6	Provider: Nurse‐led (Skinner’s teaching theory)		
	Female: 35.5%	Duration: 3 months		
	Type: Mixed^a^	Core components: Progressive program included pre‐op assessment, stoma care education (manuals, videos), teach‐back method, and postdischarge tracking via WeChat/phone.		
	Status: Temporary			

Liu and Ni [48] (China)	N: 126 (63/63)	Category: Technology‐mediated postdischarge support	Routine care	• Complications
	Age: 43.7/44.6	Provider: Nurse‐led		• WHOQOL‐BREF
	Female: 46.0%	Duration: Not specified		• HAMD and HAMA
	Type: Colostomy	Core components: Internet‐based remote continuous care. Electronic health records established. Online education, real‐time Q&A via app/platform, and regular remote monitoring.		
	Status: Permanent			

Ozel and Kaya [50] (Türkey)	N: 70 (35/35)	Category: Technology‐mediated postdischarge support	Routine care	• Complications
	Age: 55.8 vs. 59.8	Provider: Nurse‐led		• CoH‐QoL‐OQ
	Female: 37.1%	Duration: 1 month		
	Type: Mixed a	Core components: Web‐based education program. Answering questions and remote monitoring via the website.		
	Status: Not specified			

Özkaya and Harputlu [44] (Turkey)	N: 60 (30/30)	Category: Technology‐mediated postdischarge support	Routine care	• Complications
	Age: 58.5/64.2	Provider: Nurse‐led		• SSES
	Female: 25.0%	Duration: 2 months		• OAI‐23
	Type: Mixed^a^	Core components: Video‐conference education postdischarge. Scheduled video calls within the 1st to 4th weeks covering stoma care, complications, and nutrition		• Readmission
	Status: Mixed^b^			

Su et al. [37] (China)	N: 107 (50/57)	Category: Comprehensive perioperative support	Routine care	• Complications
	Age: 57.0/59.1	Provider: ET‐led (Bandura’s self‐efficacy theory)		• Stoma‐QoL
	Female: 37.4%	Duration: 3 months		• SSES
	Type: Mixed^a^	Core components: Evidence‐based continuing care bundle. Pre‐op education, post‐op skills training, and 3‐month follow‐up via structured phone follow‐up.		• Satisfaction
	Status: Temporary			

Wang et al. [49] (China)	N: 200 (100/100)	Category: Comprehensive perioperative support	Routine care	• Complications
	Age: Not specified	Provider: Nurse‐led (Transtheoretical Model)		• ASAS‐R‐C
	Female: 48.5%	Duration: 1 month		• Satisfaction
	Type: Not specified	Core components: Stage‐tailored health education program: enterostomy education skirt; 3D interactive app; VR training system; complication models; multichannel online/offline support.		
	Status: Permanent			

Wang et al. [43] (China)	N: 120 (60/60)	Category: Technology‐mediated postdischarge support	Routine care	• Complications
	Age: 51.3/50.4	Provider: MDT		• Stoma‐QoL
	Female: 40.0%	Duration: 3 months		• ESCA
	Type: Colostomy	Core components: Visual health education combined with peer education. Educational videos, WeChat interaction, and peer support sharing sessions.		• OAS
	Status: Permanent			• Satisfaction

Wen et al. [26] (China)	N: 92 (47/45)	Category: Comprehensive perioperative support	Routine care	• Self‐efficacy (Subscale)
	Age: 52.8 (total)	Provider: Nurse‐led (Transtheoretical model)		
	Female: 40.2%	Duration: 6 months		
	Type: Colostomy	Core components: Stage‐matched education and support (precontemplation to maintenance) delivered via face‐to‐face sessions.		
	Status: Permanent			

Xia [39] (China)	N: 155 (81/74)	Category: Comprehensive perioperative support	Routine care	• Complications
	Age: Not specified	Provider: ET‐led		• Stoma‐QoL
	Female: 46.5%	Duration: 3 months		• SSES
	Type: Colostomy	Core components: Stoma care education, real‐time consultations (WeChat and telephone), and a designated family caregiver to assist home care and provide feedback.		• STAI
	Status: Permanent			• Satisfaction

Shujuan 2018 [40] (China)	N: 48 (20/28)	Category: Comprehensive perioperative support	Routine care	• EORTC QLQ‐C30
	Age: 60.9/61.1	Provider: Nurse‐led (Self‐Efficacy Theory)		• SSES
	Female: 31.3%	Duration: 3 months		
	Type: Colostomy	Core components: Self‐efficacy enhancing intervention. Direct/alternative experience, psychological support, and visits or calls.		
	Status: Permanent			

Yilmazer et al. [42]. (Turkey)	N: 60 (30/30)	Category: Comprehensive perioperative support	Routine care	• SF‐36
	Age: 54.9/59.1	Provider: Nurse‐led (Information–Motivation–Behavioral Skills model)		• BAF
	Female: 53.3%	Duration: 3 months		
	Type: Mixed^a^	Core components: Postsurgery education, handbook, positive feedback, and counseling at 1 m and 3 m postdischarge.		
	Status: Mixed^b^			

Zhang et al. [38] (China)	N: 103 (52/51)	Category: Technology‐mediated postdischarge support	Routine care	• Complications
	Age: 52.9/55.3	Provider: ET‐led (Bandura’s Social Learning Theory)		• SSES
	Female: 35.0%	Duration: 1 month		• OAS
	Type: Colostomy	Core components: Two structured telephones (2w and 4w postdischarge) focusing on assessment, problem solving, and psychosocial support.		• Satisfaction
	Status: Permanent			

Zhang et al. [23] (China)	N: 108 (55/53)	Category: Comprehensive perioperative support	Routine care	• Complications
	Age: 58.6/59.4	Provider: MDT (Timing It Right)		• Stoma‐QoL
	Female: 31.9%	Duration: 6 months		• ESCA
	Type: Colostomy	Core components: Needs‐based phased education (admission, pre‐op, post‐op, discharge, home). MDT support and home visits/phone follow‐ups.		
	Status: Permanent			

Zhou et al. [36] (China)	N: 150 (75/75)	Category: Comprehensive perioperative support	Routine care	• Complications
	Age: 62.7/62.2	Provider: MDT		• GIQLI
	Female: 36.0%	Duration: 6 months		• ESCA
	Type: Mixed^a^	Core components: Discharge readiness intervention. Multistage assessment and education (from admission to home). MDT formulation of discharge plans and continuous tracking via phone.		
	Status: Mixed^b^			

*Note:* MDT, Multidisciplinary team; SAS, Self‐rating Anxiety Scale; SDS, Self‐rating Depression Scale; SF‐36, 36‐Item Short Form Survey; ESCA, The self‐care ability scale; Stoma QoL, Stoma Quality of life; WHOQOL‐BREF, World Health Organization Quality of Life Instruments; HAMD, Hamilton Depression Rating Scale; HAMA, Hamilton Anxiety Rating Scale; COH‐QOLOQ, City of Hope‐Quality of Life‐Ostomy Questionnaire; ASAS‐R‐C, Self‐Care Ability Assessment Scale Revised Chinese; EORTC QLQ‐C30, European Organization for Research and Treatment of Cancer Quality of Life Questionnaire Core 30; BAF, The Behavior Assessment Form; GIQLI, Gastrointestinal Quality of Life Index Scale.

Abbreviations: ET = enterostomal therapist, LOS = length of stays, OAI‐23 = Ostomy Adjustment Inventory‐23; OAS = Ostomy Adjustment Scale, QoL = Quality of Life, SSES = Stoma Self‐efficacy Scale, STAI = State‐Trait Anxiety Inventory.

^a^Stoma type: mixed (ileostomy, colostomy).

^b^Stoma status: mixed (temporary, permanent).

Regarding stoma characteristics, the studies included both ileostomy and colostomy patients. Specifically, eight studies focused solely on colostomy [23, 26, 38–40, 43, 45, 48], nine included mixed stoma types [24, 36, 37, 41, 42, 44, 46, 47, 50], and one did not specify the type [49]. In terms of stoma permanence, eight studies recruited patients with permanent stomas [23, 26, 38–40, 43, 48, 49], three involved temporary stomas [37, 45, 46], and four included both [36, 41, 42, 44]; the remaining three studies did not report these data [24, 47, 50].

Given differences in delivery mode and intensity, continuous care strategies were classified into two typologies. Comprehensive perioperative support (*n* = 11) [23, 24, 26, 36, 37, 39–42, 46, 49] represented the most robust model, characterized by the integration of preoperative or early postoperative education with structured, longitudinal follow‐up extending into the postdischarge period. Technology‐mediated postdischarge support (*n* = 7) [38, 43–45, 47, 48, 50] primarily leveraged digital health technologies—including telephone consultations, mobile applications, and online platforms—as the central mechanism for remote monitoring (Supporting Table 3). Regarding implementation characteristics, nine studies grounded their interventions in a theoretical framework [23, 26, 37, 38, 40–42, 46, 49]. The majority were nurse‐led (*n* = 10) [26, 40–42, 44, 46–50], while five employed an MDT approach [23, 24, 36, 43, 45], and three were delivered by enterostomal therapists [37–39].

Across the included trials, usual care generally referred to the routine care provided by each participating institution, such as routine perioperative or inpatient stoma care, discharge education, and routine outpatient or telephone follow‐up when available. None of the comparator groups included a structured, proactive, and longitudinal continuous care program comparable to the intervention group.

### 3.3. Risk of Bias

Risk‐of‐bias assessments for patient‐reported and objective outcomes are visually summarized in Supporting Figures 1 and 2, respectively. Seventeen studies reported patient‐reported subjective outcomes [23, 24, 26, 36–45, 47–50]; among these, only two were classified as low risk [41, 49], while eight had some concerns, and seven were judged as high risk. Fourteen studies reported objective complication outcomes [23, 24, 36–39, 43–50]; of these, five were judged to have a low risk of bias [23, 24, 44, 46, 49], five raised some concerns [36–39, 43], and four were assessed as high risk [45, 47, 48, 50].

Regarding the randomization process, 14 studies provided adequate description of allocation concealment [23, 24, 26, 36–44, 46, 49], whereas four studies lacked sufficient detail on sequence generation or allocation procedures [45, 47, 48, 50]. Nevertheless, baseline characteristics were balanced across groups in all included trials. Performance and detection bias presented notable challenges; due to the nature of the interventions, blinding of participants and personnel was inherently infeasible. Consequently, blinding of outcome assessors was the primary strategy to mitigate bias, which was implemented in eight studies [23, 24, 26, 36–38, 41, 49]. However, nine studies failed to report blinding procedures [40, 42–48, 50]. Attrition bias was generally low, with 11 studies employing intention‐to‐treat (ITT) analysis [23, 26, 39, 42–45, 47–50] and dropout rates in reporting studies not exceeding 17% [24, 36–38, 40, 41, 46]. Finally, concerns regarding selective reporting were identified in most studies, primarily due to the absence of prospectively published protocols.

### 3.4. Certainty of the Evidence

Based on the GRADE assessment, the certainty of evidence varied across outcomes (Table 2). Complications, QoL, and self‐efficacy outcomes were rated as low, and evidence for self‐care, ostomy adjustment, anxiety, and depression were downgraded to very low certainty.

**TABLE 2:** GRADE evidence profile.

Certainty assessment								No of patients		Effect		Certainty
Outcome	No of studies	Study design	Risk of bias	Inconsistency	Indirectness	Imprecision	Other considerations	Experiment	Control	Relative (95% CI)	Effect sizes (95% CI)	
Complications	11	RCT	Serious^a^	Not serious^c^	Not serious^e^	Not serious^f^	Publication bias strongly suspected^h^	56/649 (8.6%)	179/650 (27.5%)	RR 0.34 (0.26–0.45)	‐	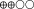 Low
Quality of life	9	RCT	Serious^a^	Serious^d^	Not serious^e^	Not serious^f^	—^i^	495	499	—	SMD 1.55 SD higher (0.91 higher to 2.19 higher)	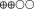 Low
Self‐care ability	7	RCT	Very serious^b^	Serious^d^	Not serious^e^	Not serious^f^	—^i^	420	419	—	SMD 1.57 SD higher (0.82 higher to 2.32 higher)	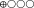 Very Low
Self‐efficacy	7	RCT	Serious^a^	Serious^d^	Not serious^e^	Not serious^f^	—^i^	358	360	—	SMD 1.29 SD higher (0.64 higher to 1.94 higher)	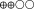 Low
Ostomy adjustment	3	RCT	Serious^a^	Serious^d^	Not serious^e^	Serious^g^	—^i^	142	141	—	SMD 1.39 SD higher (0.28 higher to 2.5 higher)	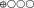 Very low
Anxiety	4	RCT	Very serious^b^	Serious^d^	Not serious^e^	Not serious^f^	—^i^	228	221	—	SMD 2.14 SD lower (3.35 lower to 0.93 lower)	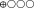 Very Low
Depression	3	RCT	Very serious^b^	Serious^d^	Not serious^e^	Serious^g^	—^i^	147	147	—	SMD 2.07 SD lower (3.89 lower to 0.25 lower)	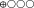 Very low

Abbreviations: CI = confidence interval, RCT = randomized controlled trial, RR = risk ratio, SMD = standardized mean difference.

^a^Most of the included studies were rated as some concerns or high risk.

^b^Most of the included studies (≥ 50%) were rated as high risk.

^c^ *I* ^2^ = 0.

^d^The evidence was downgraded due to substantial unexplained heterogeneity (*I* ^2^ > 75%).

^e^No indirectness in terms of population, intervention, comparison, or outcomes.

^f^Total sample size > 300.

^g^Total sample size < 300.

^h^The funnel plot suggests the potential presence of publication bias.

^i^Publication bias could not be assessed due to an insufficient number of studies (< 10).

### 3.5. Effects of Continuous Care Intervention for Adults With Enterostomy

#### 3.5.1. Clinical Outcomes: Stoma‐Related Complications

Eleven studies [23, 24, 36–38, 44–49], including 1299 participants, provided data suitable for meta‐analysis of stoma‐related complications. This composite outcome was defined by the original studies and generally covered stoma‐ or pouch‐related adverse events, including peristomal dermatitis, skin irritation, leakage, infection, bleeding, edema, prolapse, retraction, stenosis, and related complications where specified. Study‐specific definitions and assessment methods are provided in Supporting Table 2. Pooled analysis demonstrated that continuous care interventions significantly reduced the risk of complications compared to usual care (RR = 0.34; 95% CI: 0.26–0.45; low quality of evidence) (Supporting Figure. 3A), with negligible heterogeneity (*I* ^2^ = 0%). Leave‐one‐out sensitivity analysis yielded stable pooled estimates (RR range 0.32–0.36; *I* ^2^ = 0%), suggesting that the overall effect was not unduly influenced by any single study (Supporting Figure 3B). Restriction to eight studies with a low‐to‐moderate risk of bias (*n* = 8) maintained a similar protective effect (RR = 0.35; 95% CI: 0.24–0.49; *I* ^2^ = 13.3%) (Supporting Figure 3C).

In subgroup analyses stratified by intervention category, both comprehensive perioperative support and technology‐mediated postdischarge support were associated with reduced complication rates, with the former showing a numerically larger effect. Nevertheless, the between‐group difference was not statistically significant (*p* = 0.43) (Figure 2A). When stratified by delivery personnel, interventions led by nurses, MDT, and enterostomal therapists all yielded favorable point estimates, with MDT‐led care demonstrating the greatest numerical reduction; similarly, no significant subgroup differences were observed (*p* = 0.29) (Figure 2B).

**FIGURE 2:**
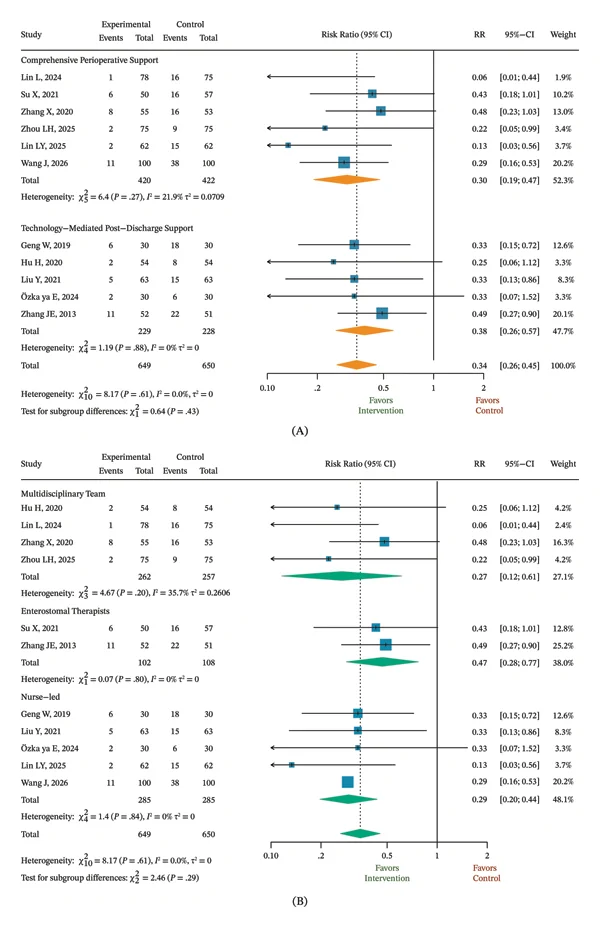
Forest plot: Effects of continuous care interventions on the incidence of stoma‐related complications across different intervention categories (A) and delivery personnel (B).

Assessment of publication bias revealed noticeable asymmetry in the visual inspection of the funnel plot, a finding corroborated by Egger’s test (*t* = −2.876, *p* = 0.018), indicating potential small‐study effects (Supporting Figure 3D).

#### 3.5.2. Psychosocial Well‐Being Outcomes

QoL outcomes were synthesized from nine studies [23, 24, 36, 37, 39–43] involving 994 participants. Measurement tools included the stoma‐QoL scale (*n* = 6) [23, 24, 37, 39, 41, 43] and generic instruments (SF‐36, EORTC QLQ‐C30, GIQLI; *n* = 3) [36, 40, 42]. Pooled analysis revealed that continuous care interventions significantly improved QoL scores compared to usual care (SMD = 1.55; 95% CI: 0.91–2.19; I^2^ = 93.1%; low quality of evidence) (Supporting Figure 4A). Leave‐one‐out sensitivity analysis produced stable pooled estimates (SMD range 1.30–1.68), indicating that the positive effect was not driven by any single outlier (Supporting Figure 4B). Restriction to seven studies with a low‐to‐moderate risk of bias maintained a similarly large effect (SMD = 1.58; 95% CI: 0.81–2.36; *I* ^2^ = 94.3%) (Supporting Figure 4C).

A total of seven studies [23, 36, 41–43, 45, 49] (*n* = 839) assessed self‐care ability using four different instruments (ESCA, ASAS‐R‐C, BAF, and the Ostomy Self‐care Ability Scale). Pooled analysis showed that continuous care interventions significantly improved self‐care ability (SMD = 1.57; 95% CI: 0.82–2.32; *I* ^2^ = 95.3%; very low quality of evidence) (Supporting Figure 5A). Leave‐one‐out sensitivity analysis demonstrated stable effect estimates (SMD range 1.30–1.82), indicating that no individual study substantially altered the overall effect (Supporting Figure 5B). After restricting to low‐bias studies (*n* = 4), the intervention effect remained favorable (SMD = 1.53; 95% CI: 0.31 to 2.76; *I* ^2^ = 97%) (Supporting Figure 5C).

Seven studies [24, 26, 37–40, 44] (*n* = 718) examined stoma‐related self‐efficacy using the SSES or a self‐efficacy subscale. Meta‐analysis showed a significant improvement in self‐efficacy (SMD = 1.29; 95% CI: 0.64–1.94; *I* ^2^ = 91.9%; low quality of evidence) (Supporting Figure 6A). Leave‐one‐out sensitivity analysis yielded consistent estimates (SMD range 1.12–1.50), indicating that the pooled effect was not driven by any single study (Supporting Figure 6B). When restricting to low‐to‐moderate risk‐of‐bias studies (*n* = 5), the direction and magnitude of effect remained favorable (SMD = 1.26; 95% CI: 0.51–2.00; *I* ^2^ = 92.5%) (Supporting Figure 6C).

Three studies [38, 43, 44] (*n* = 283) assessed ostomy adjustment using the OAS or the OAI‐23. Pooled analysis indicated that continuous care interventions improved ostomy adjustment (SMD = 1.39; 95% CI: 0.28–2.50; *I* ^2^ = 91.7%; very low quality of evidence) (Supporting Figure 7A). Leave‐one‐out sensitivity analysis showed that omission of one study [43] resulted in a CI crossing the line of no effect (Supporting Figure 7B), highlighting the need for cautious interpretation. After excluding one study at a high risk of bias, the direction of effect remained favorable (SMD = 1.83; 95% CI: 0.55–3.11; *I* ^2^ = 90.5%) (Supporting Figure 7C).

Four [39, 45, 47, 48] studies (*n* = 449) evaluated anxiety outcomes using the SAS, HAMA, and STAI. Pooled analysis showed that continuous care significantly reduced anxiety compared with usual care (SMD = −2.14; 95% CI: −3.35 to −0.93; *I* ^2^ = 91.4%; very low quality of evidence) (Supporting Figure 8A). Leave‐one‐out sensitivity analyses produced effect estimates ranging from −2.48 to −1.55, with CIs consistently excluding the null, indicating that the overall effect was not driven by any single study (Supporting Figure 8B). Given that three of the four studies were rated at a high risk of bias and only one study was rated as some concerns, conducting a restricted analysis excluding high‐risk studies was not meaningful and was therefore not undertaken.

Three studies [45, 47, 48] (*n* = 294) reported depression outcomes using SDS or HAMD. Continuous care was associated with significantly lower depression scores (SMD = −2.07; 95% CI: −3.89 to −0.25; *I* ^2^ = 96.0%; very low quality of evidence) (Supporting Figure 9A). Sensitivity analyses showed pooled SMDs ranging from −2.79 to −1.21, all remaining on the beneficial side of the null, suggesting a stable direction of effect (Supporting Figure 9B). Because all included studies were rated at a high risk of bias, a sensitivity analysis excluding high‐risk studies was not performed.

Seven studies [37–39, 43, 45, 47, 49] evaluated patient satisfaction. Because measurement tools, scoring formats, and reporting methods varied substantially across studies, a quantitative meta‐analysis was not feasible. Instead, a narrative synthesis was conducted. Across all seven studies, continuous care interventions were associated with higher satisfaction levels than usual care, with statistically significant differences reported in each study (all *p* < 0.05). These findings suggest a favorable effect on patient‐reported satisfaction, while remaining limited by heterogeneity in measurement approaches.

#### 3.5.3. Healthcare Utilization

Because definitions, measurement methods, and reporting formats for healthcare utilization differed across studies, quantitative pooling was not conducted, and the results were narratively synthesized. Three studies [24, 41, 44] reported unplanned readmission rates, and two studies [24, 41] reported length of hospital stay. No eligible RCT reported healthcare costs. Across studies, continuous care was associated with fewer unplanned readmissions. Lin et al. [24] observed markedly lower readmissions at 1 and 3 months in the experimental group (2 and 1) compared with those in the control group (12 and 8) (*p* < 0.05), and Özkaya et al. [44] similarly reported fewer readmissions at 2 months (3 vs. 9; *p* < 0.05). Li et al. [41] also found a reduced readmission proportion (39% vs. 61%). Length‐of‐stay findings were mixed: Lin et al. [24] reported no difference (median 10 days in both groups), whereas Li et al. [41] found shorter stays in the experimental group (median 15 days [IQR 12–20] vs. 18 days [IQR 15–22]).

## 4. Discussion

To our knowledge, this study represents the first systematic review and meta‐analysis exclusively synthesizing RCTs to evaluate the multifaceted impact of continuous care for adults with enterostomy. By analyzing data from 18 RCTs involving 1926 participants, our findings suggest that extending professional support beyond the hospital setting improves clinical outcomes, psychosocial well‐being, and selected healthcare utilization outcomes. In this review, continuous care was considered a complex transitional‐care strategy, unified by the shared aim of maintaining professional support across the hospital‐to‐home transition. Specifically, continuous care interventions reduced the risk of stoma‐related complications by 66% compared with usual care. These interventions also improved QoL, self‐care ability, self‐efficacy, and ostomy adjustment, and reduced anxiety and depression. However, the patient‐reported outcomes should be interpreted cautiously because they showed substantial heterogeneity and were generally supported by low or very low certainty evidence. Although heterogeneity precluded quantitative pooling for healthcare utilization, narrative synthesis indicated fewer unplanned hospital readmissions in the intervention groups. Collectively, these results support the clinical relevance of continuous care while highlighting the need for more standardized, higher‐quality trials to determine which components, delivery modes, personnel, intensity, and duration are most effective and scalable in practice.

A salient finding of this review is the reduction in stoma‐related complications (RR = 0.34) associated with continuous care. This protective effect was consistent, characterized by negligible heterogeneity (*I* ^2^ = 0%) and stability across sensitivity analyses. The consistency and precision of these estimates strengthen confidence in this clinical outcome and compare favorably with earlier reviews, which reported greater variability due to mixed study designs and nonrandomized evidence [28]. The beneficial effect may be explained by early competency acquisition, whereby structured perioperative education and repeated follow‐up enable patients to master essential technical skills, such as appliance fitting, peristomal skin protection, and early recognition of warning signs, thereby preventing minor skin issues from progressing into more serious complications. Furthermore, the psychological reassurance provided by continuous professional access may enhance adherence to care protocols, contributing to safer postoperative recovery [11, 12].

In terms of intervention modalities, comprehensive perioperative support showed the largest nominal reduction in complication risk. By initiating skills training preoperatively or early postoperatively, this model allows patients and families sufficient time to internalize care techniques before discharge. Similarly, regarding delivery personnel, MDT‐led interventions produced the greatest nominal effect, possibly reflecting coordinated input from surgeons, enterostomal therapists, nurses, and nutritionists. However, differences between subgroups did not reach statistical significance. Accordingly, these numerical differences do not establish the superiority of MDT‐led or comprehensive models over other forms of continuous care. The lack of statistical significance may stem from limited statistical power in subgroup stratifications. Future large‐scale trials are needed to determine whether intervention modality or delivery personnel modifies effectiveness.

This review also suggests favorable effects of continuous care on psychosocial outcomes. Nevertheless, interpretation of these findings requires caution, given the substantial heterogeneity, frequent high risk of bias, and low or very low certainty of evidence in the patient‐reported outcome studies. Our meta‐analysis demonstrated improvements in QoL (SMD = 1.55), self‐care ability (SMD = 1.57), self‐efficacy (SMD = 1.29), and ostomy adjustment (SMD = 1.39), but these large SMDs should not be interpreted as definitive estimates of intervention magnitude. Rather, they indicate a consistently favorable direction of effect across studies using different instruments, intervention intensities, and follow‐up schedules. Patients with enterostomy often experience social isolation and role restriction due to fear of leakage and altered body image. Continuous care may target these stressors by improving appliance management, dietary guidance, and access to professional feedback, thereby supporting social participation and self‐management [23, 36]. Interpreted through Bandura’s Social Learning Theory [51], repeated guidance and feedback may provide “mastery experiences” that strengthen self‐efficacy and facilitate better ostomy adjustment [26, 37].

Furthermore, the observed reductions in anxiety (SMD = −2.14) and depression (SMD = −2.07) point to a potentially important role for continuous care in survivorship support. The immediate postdischarge period is often a peak period for psychological distress because patients lose the continuous supervision available in hospital. Scheduled access to professional advice through telephone, home visits, or telehealth may reduce anticipatory anxiety about leakage, skin complications, and self‐care failure. This sustained professional connection may validate patients’ emotional experiences and reduce the likelihood that transient distress becomes persistent psychological morbidity [15, 52]. Patient satisfaction was also consistently higher in intervention groups [37, 47], probably reflecting the perceived accessibility and responsiveness of continuous care models. However, because anxiety, depression, and satisfaction outcomes were limited by study quality, small numbers of trials, and heterogeneous measures, these findings should be considered hypothesis‐generating and require confirmation in rigorously designed trials.

In addition, this review provides insights for health policy and resource allocation by extending the scope of analysis to healthcare utilization. The lower readmission rates reported across three studies [24, 41, 44] suggest potential preventive value for continuous care. Mechanistically, this benefit may stem from empowering patients to resolve minor issues—such as early dehydration or mild skin irritation—in the home setting, thereby averting the clinical deterioration that typically triggers emergency rehospitalization [53, 54]. Evidence regarding length of stay was mixed, and no eligible RCT reported healthcare costs. The lack of significant LOS reduction in some trials [24] may reflect a “floor effect” driven by the widespread adoption of ERAS protocols, which have already minimized baseline hospitalization times. Future research should incorporate rigorous cost‐utility analyses to determine the long‐term return on investment [55, 56].

Recent reviews have examined educational programs, self‐care, or adaptation across broader nursing interventions for people with stomas [57–59], while earlier syntheses focused on continuing care, QoL, telemedicine, or eHealth [22, 27–30]. This review differs by restricting eligibility to RCTs in adults with enterostomy and to interventions that provide structured professional support across the hospital‐to‐home transition. The classification of intervention typologies and delivery personnel also helps clarify how a shared transitional‐care goal may be implemented through different models. In addition, separate RoB 2 and GRADE assessments for objective and patient‐reported outcomes support a more nuanced interpretation: evidence for reducing stoma‐related complications appears more consistent, whereas the large psychosocial effects remain promising but less certain. By also considering readmissions and length of stay while identifying the absence of eligible cost evidence, this review defines priorities for future implementation and health‐economic research.

### 4.1. Limitations

Several limitations of this review warrant consideration when interpreting the findings. First, regarding methodological quality, the majority of the 18 included RCTs were rated as having “some concerns” or “high risk of bias.” This was predominantly driven by the inherent difficulty of blinding participants and personnel in behavioral nursing interventions, which may introduce performance and detection bias. Second, the certainty of evidence, assessed via GRADE, ranged from low to very low for most outcomes. This grading reflects limitations in study design, imprecision, and inconsistency, suggesting that the true effects may differ from our estimates and precluding strong clinical recommendations for several outcomes. Third, potential publication bias was identified for the primary outcome of stoma‐related complications, indicated by funnel plot asymmetry. This suggests a possible overestimation of the intervention’s protective effect due to the nonpublication of small studies with negative results. Fourth, substantial statistical heterogeneity persisted for most psychosocial outcomes despite the use of random‐effects models and sensitivity analyses. This variability may reflect clinical diversity in intervention protocols, differences in measurement instruments, and variation in follow‐up periods. Additionally, the content of usual care differed across trials and may have contributed to clinical heterogeneity. Because the secondary meta‐analyses included only 3–9 studies with sparse and uneven distributions across candidate covariates, subgroup or moderator analyses and meta‐regression were restricted to the primary outcome. This protocol deviation limited further exploration of heterogeneity in the secondary outcomes. Finally, most included studies were conducted in China, with three from Turkey, restricting the generalizability of our results. Given regional differences in healthcare systems, stoma nursing roles, reimbursement structures, and postdischarge support models, further RCTs from a broader range of countries are required to confirm the applicability of continuous care models across healthcare contexts.

## 5. Conclusion

This systematic review and meta‐analysis of 18 RCTs suggests that continuous care may be a valuable strategy for improving postdischarge outcomes in adults with enterostomy. By bridging the transition from hospital to home, continuous care reduced the risk of stoma‐related complications and improved psychosocial outcomes, including QoL, self‐care ability, self‐efficacy, ostomy adjustment, anxiety, and depression. The observed reduction in unplanned hospital readmissions also suggests potential value for healthcare systems. However, these conclusions should be interpreted in light of the substantial heterogeneity, methodological limitations, and low or very low certainty of evidence for many patient‐reported outcomes. Current findings support the development of structured, early, and patient‐centered continuous care programs, ideally with clear active components and access to specialist or MDT expertise. Future research should prioritize large, multicenter RCTs with standardized intervention reporting, longer follow‐up, core outcome measures, and robust health‐economic evaluation to determine which continuous care models are most effective, generalizable, and sustainable.

## 6. Implications for Nursing Management

Nurse managers should consider developing structured hospital‐to‐home pathways with clear governance, defined professional roles, competency‐based training, and multidisciplinary coordination. These pathways should incorporate discharge preparation, self‐care readiness assessment, planned follow‐up supported by reliable infrastructure, and access to specialist or multidisciplinary support, with staffing and resource allocation aligned with local capacity. Programs should monitor stoma‐related complications, patient‐reported outcomes, readmissions, and resource use.

## Author Contributions

Ru Yang: conceptualization, methodology, validation, funding acquisition, writing–original draft, and writing–review and editing. Zeyao Shi: conceptualization, validation, and writing–original draft. Yingxin Li: conceptualization, validation, and writing–original draft. Xi Huang: conceptualization, validation, and writing–original draft. Xia Li: conceptualization, validation, and writing–original draft. Xushu Chen: conceptualization, validation, and writing–original draft; Qiong Chen: conceptualization, data curation, and writing–original draft. Yanling Hu: conceptualization, data curation, and writing–original draft. Yuan Li: conceptualization, methodology, supervision, project administration, funding acquisition, and writing–review and editing. Xiaowen Li: conceptualization, methodology, supervision, and writing–review and editing.

## Funding

The work was supported by grants from the Sichuan Provincial Maternal and Child Health Association Research Project (Grant No. 2024FX13), the Sichuan Province Nursing Association (Grant No. H23020), the Sichuan Medical Association (Grant No. Q2024011), and the Chengdu Municipal Bureau of Science and Technology (Grant No. 2024‐YF05‐00503‐SN).

## Disclosure

The funders had no role in study design, data collection and analysis, decision to publish, or preparation of the manuscript.

## Ethics Statement

No ethics approval was required for this systematic review and meta‐analysis. Because it used only data from previously published studies and did not involve direct contact with participants or collection of identifiable personal information.

## Conflicts of Interest

The authors declare no conflicts of interest.

## Supporting Information

Additional supporting information can be found online in the Supporting Information section.

## Supporting information


**Supporting Information** Supporting information 1. PRISMA 2020 Statement. Supporting information 2. Search strategy (Updated September 1, 2026). Supporting Table 1. Reason for exclusion of studies screened at full text. Supporting Table 2. Characteristics and quality appraisal of the included studies (*n* = 18). Supporting Table 3. Intervention classification for continuous care in stoma patients. Supporting Figure 1. Risk‐of‐bias assessment (the incidence of stoma‐related complications) of including studies. Supporting Figure 2. Risk‐of‐bias assessment (patient‐reported subjective outcomes) of including studies. Supporting Figure 3. Forest plot of the effect of continuous care on stoma‐related complications (A); sensitivity analysis (B); analysis after excluding studies with a high risk of bias (C); and funnel plot for publication bias assessment (*D*). Supporting Figure 4. Forest plot of the effect of continuous care on QoL (A); sensitivity analysis (B); and analysis after excluding studies with a high risk of bias (C). Supporting Figure 5. Forest plot of the effect of continuous care on self‐care ability (A); sensitivity analysis (B); and analysis after excluding studies with a high risk of bias (C). Supporting Figure 6. Forest plot of the effect of continuous care on self‐efficacy (A); sensitivity analysis (B); and analysis after excluding studies with a high risk of bias (C). Supporting Figure 7. Forest plot of the effect of continuous care on ostomy adjustment (A); sensitivity analysis (B); and analysis after excluding studies with a high risk of bias (C). Supporting Figure 8. Forest plot of the effect of continuous care on anxiety (A) and sensitivity analysis (B). Supporting Figure 9. Forest plot of the effect of continuous care on depression (A) and sensitivity analysis (B).

## Data Availability

The data that support the findings of this study are available in the supporting information of this article.
