# Supplementary material for: Effects of Continuous Care on Clinical Outcomes, Psychosocial Well‐Being, and Healthcare Utilization in Adults With Enterostomy: A Systematic Review and Meta‐Analysis of Randomized Controlled Trials
Source: J Nurs Manag. 2026 Oct 19;2026:1161340. doi: 10.1155/jonm/1161340 (PMC13678411; doi:10.1155/jonm/1161340)
Supplement: Supplementary file 1 [file JONM-2026-1161340-s001.docx]

**Supplementary Materials**

[**Content**	1](#_Toc217153856)

[**Supplementary Material 1** PRISMA 2020 Statement	2](#_Toc217153857)

[**Supplementary Material 2** Search strategy (Updated Sep 1, 2025)	4](#_Toc217153858)

[**Supplementary Table 1.** Reason for exclusion of studies screened at full text	8](#_Toc217153859)

[**Supplementary Table 2.** Characteristics and quality appraisal of the included studies (n = 18).	12](#_Toc217153860)

[**Supplementary Table 3.** Intervention Classification for Continuous Care in Stoma Patients	23](#_Toc217153861)

[**Supplementary Figure 1.** Risk of bias assessment (the incidence of stoma-related complications) of included studies	24](#_Toc217153862)

[**Supplementary Figure 2.** Risk of bias assessment (patient-reported subjective outcomes) of included studies	24](#_Toc217153863)

[**Supplementary Figure 3.** Forest plot of the effect of continuous care on stoma-related complications (A); sensitivity analysis (B); analysis after excluding studies with high risk of bias (C); and funnel plot for publication bias assessment (D).	25](#_Toc217153864)

[**Supplementary Figure 4.** Forest plot of the effect of continuous care on QoL (A); sensitivity analysis (B); and analysis after excluding studies with high risk of bias (C).	27](#_Toc217153865)

[**Supplementary Figure 5.** Forest plot of the effect of continuous care on self-care ability (A); sensitivity analysis (B); and analysis after excluding studies with a high risk of bias (C).	28](#_Toc217153866)

[**Supplementary Figure 6.** Forest plot of the effect of continuous care on self-efficacy (A); sensitivity analysis (B); and analysis after excluding studies with a high risk of bias (C).	29](#_Toc217153867)

[**Supplementary Figure 7.** Forest plot of the effect of continuous care on ostomy adjustment (A); sensitivity analysis (B); and analysis after excluding studies with a high risk of bias (C).	30](#_Toc217153868)

[**Supplementary Figure 8.** Forest plot of the effect of continuous care on anxiety (A); and sensitivity analysis (B).	31](#_Toc217153869)

[**Supplementary Figure 9.** Forest plot of the effect of continuous care on depression (A); and sensitivity analysis (B).	32](#_Toc217153870)

**Supplementary Material 1** PRISMA 2020 Statement

| **Section and Topic** | **Item #** | **Checklist item** | **Location where item is reported** |
| --- | --- | --- | --- |
| **TITLE** | | |  |
| Title | 1 | Identify the report as a systematic review. | 1 |
| **ABSTRACT** | | |  |
| Abstract | 2 | See the PRISMA 2020 for Abstracts checklist. | 2-3 |
| **INTRODUCTION** | | |  |
| Rationale | 3 | Describe the rationale for the review in the context of existing knowledge. | 3-5 |
| Objectives | 4 | Provide an explicit statement of the objective(s) or question(s) the review addresses. | 5 |
| **METHODS** | | |  |
| Eligibility criteria | 5 | Specify the inclusion and exclusion criteria for the review and how studies were grouped for the syntheses. | 6-7 |
| Information sources | 6 | Specify all databases, registers, websites, organisations, reference lists and other sources searched or consulted to identify studies. Specify the date when each source was last searched or consulted. | 6 |
| Search strategy | 7 | Present the full search strategies for all databases, registers and websites, including any filters and limits used. | Sup Material 2 |
| Selection process | 8 | Specify the methods used to decide whether a study met the inclusion criteria of the review, including how many reviewers screened each record and each report retrieved, whether they worked independently, and if applicable, details of automation tools used in the process. | 7-8 |
| Data collection process | 9 | Specify the methods used to collect data from reports, including how many reviewers collected data from each report, whether they worked independently, any processes for obtaining or confirming data from study investigators, and if applicable, details of automation tools used in the process. | 7-8 |
| Data items | 10a | List and define all outcomes for which data were sought. Specify whether all results that were compatible with each outcome domain in each study were sought (e.g. for all measures, time points, analyses), and if not, the methods used to decide which results to collect. | 7 |
|  | 10b | List and define all other variables for which data were sought (e.g. participant and intervention characteristics, funding sources). Describe any assumptions made about any missing or unclear information. | 8 |
| Study risk of bias assessment | 11 | Specify the methods used to assess risk of bias in the included studies, including details of the tool(s) used, how many reviewers assessed each study and whether they worked independently, and if applicable, details of automation tools used in the process. | 8 |
| Effect measures | 12 | Specify for each outcome the effect measure(s) (e.g. risk ratio, mean difference) used in the synthesis or presentation of results. | 9 |
| Synthesis methods | 13a | Describe the processes used to decide which studies were eligible for each synthesis (e.g. tabulating the study intervention characteristics and comparing against the planned groups for each synthesis (item #5)). | 9 |
|  | 13b | Describe any methods required to prepare the data for presentation or synthesis, such as handling of missing summary statistics, or data conversions. | 9 |
|  | 13c | Describe any methods used to tabulate or visually display results of individual studies and syntheses. | 9 |
|  | 13d | Describe any methods used to synthesize results and provide a rationale for the choice(s). If meta-analysis was performed, describe the model(s), method(s) to identify the presence and extent of statistical heterogeneity, and software package(s) used. | 9 |
|  | 13e | Describe any methods used to explore possible causes of heterogeneity among study results (e.g. subgroup analysis, meta-regression). | 9 |
|  | 13f | Describe any sensitivity analyses conducted to assess robustness of the synthesized results. | 9 |
| Reporting bias assessment | 14 | Describe any methods used to assess risk of bias due to missing results in a synthesis (arising from reporting biases). | 9 |
| Certainty assessment | 15 | Describe any methods used to assess certainty (or confidence) in the body of evidence for an outcome. | 9 |
| **RESULTS** | | |  |
| Study selection | 16a | Describe the results of the search and selection process, from the number of records identified in the search to the number of studies included in the review, ideally using a flow diagram. | 10 |
|  | 16b | Cite studies that might appear to meet the inclusion criteria, but which were excluded, and explain why they were excluded. | Sup Table 1 |
| Study characteristics | 17 | Cite each included study and present its characteristics. | 10-17 |
| Risk of bias in studies | 18 | Present assessments of risk of bias for each included study. | 18 |
| Results of individual studies | 19 | For all outcomes, present, for each study: (a) summary statistics for each group (where appropriate) and (b) an effect estimate and its precision (e.g. confidence/credible interval), ideally using structured tables or plots. | 20-24 |
| Results of syntheses | 20a | For each synthesis, briefly summarise the characteristics and risk of bias among contributing studies. | 10-17 |
|  | 20b | Present results of all statistical syntheses conducted. If meta-analysis was done, present for each the summary estimate and its precision (e.g. confidence/credible interval) and measures of statistical heterogeneity. If comparing groups, describe the direction of the effect. | 20-24 |
|  | 20c | Present results of all investigations of possible causes of heterogeneity among study results. | 20-24 |
|  | 20d | Present results of all sensitivity analyses conducted to assess the robustness of the synthesized results. | 20-24 |
| Reporting biases | 21 | Present assessments of risk of bias due to missing results (arising from reporting biases) for each synthesis assessed. | 19 |
| Certainty of evidence | 22 | Present assessments of certainty (or confidence) in the body of evidence for each outcome assessed. | 18-19 |
| **DISCUSSION** | | |  |
| Discussion | 23a | Provide a general interpretation of the results in the context of other evidence. | 24 |
|  | 23b | Discuss any limitations of the evidence included in the review. | 27 |
|  | 23c | Discuss any limitations of the review processes used. | 27 |
|  | 23d | Discuss implications of the results for practice, policy, and future research. | 28 |
| **OTHER INFORMATION** | | |  |
| Registration and protocol | 24a | Provide registration information for the review, including register name and registration number, or state that the review was not registered. | 5-6 |
|  | 24b | Indicate where the review protocol can be accessed, or state that a protocol was not prepared. | 5-6 |
|  | 24c | Describe and explain any amendments to information provided at registration or in the protocol. | 5-6 |
| Support | 25 | Describe sources of financial or non-financial support for the review, and the role of the funders or sponsors in the review. | 29 |
| Competing interests | 26 | Declare any competing interests of review authors. | 29 |
| Availability of data, code and other materials | 27 | Report which of the following are publicly available and where they can be found: template data collection forms; data extracted from included studies; data used for all analyses; analytic code; any other materials used in the review. | 29-30 |

*From:* Page MJ, McKenzie JE, Bossuyt PM, Boutron I, Hoffmann TC, Mulrow CD, et al. The PRISMA 2020 statement: an updated guideline for reporting systematic reviews. BMJ 2021;372:n71. doi: 10.1136/bmj.n71. This work is licensed under CC BY 4.0. To view a copy of this license, visit <https://creativecommons.org/licenses/by/4.0/>

**Supplementary Material 2** Search strategy (Updated Sep 1, 2026)

MEDLINE (Ovid)

1	case management/ or disease management/ or (manage* adj2 (care? or case? or disease?)).tw.

2	continuity of patient care/ or aftercare/ or patient discharge/ or transitional care/ or ambulatory care/ or long-term care/ or home care services/ or home care services, hospital-based/ or home health nursing/ or home nursing/ or hospital to home transition/

3	((transition* or transfer* or continu* or coordinat* or collaborat* or seamless or integrat* or comprehensive or intermediate or extended or follow-up or postdischarge or post-discharge or posthospital* or post-hospital* or home or home-based or domiciliary or long-term or outpatient or ambulatory) adj3 (care? or service? or intervention?)).tw.

4	((interdisciplinary or multidisciplinary or interprofessional) adj3 (program* or management)).tw.

5	((patient adj2 (discharge or follow-up)) or (discharge? adj2 plan*) or (case adj2 management) or aftercare* or after care).tw.

6	monitoring, ambulatory/ or telemedicine/ or telenursing/ or remote consultation/ or mobile applications/ or internet/

7	(((ambulatory or remote or distan* or outpatient) adj3 monitor*) or (telemed* or telemed* or telehealth* or tele-health* or tele-consult* or eHealth or mHealth or Mobile Health or telecare* or telenurs* or telemonitor* or teleconsult* or tele-consult* or teleconferenc* or tele-conferenc* or telecommunicat* or telecardiol* or telephon* or telemetry or phone* or messag* or consultation* or application* or mobile* or app* or software*)).tw.

8	enterostomy/ or cecostomy/ or colostomy/ or duodenostomy/ or ileostomy/ or jejunostomy/ or surgical stomas/ or ostomy/

9	(enterostom* or cecostom* or colostom* or duodenostom* or ileostom* or jejunostom* or ostom*).tw.

10	((Surgical or colon) adj2 Stoma*).tw.

11	(exp randomized controlled trial/ or controlled clinical trial.pt. or randomized.ab. or placebo.ab. or drug therapy.fs. or randomly.ab. or trial.ab. or groups.ab.) not (exp animals/ not humans.sh.)

12	1 or 2 or 3 or 4 or 5 or 6 or 7

13	8 or 9 or 10

14	11 and 12 and 13

Embase (Ovid)

1	patient care/ or aftercare/ or hospital discharge/ or transitional care/ or ambulatory care/ or long term care/ or home care/ or hospital to home transition/

2	((transition* or transfer* or continu* or coordinat* or collaborat* or seamless or integrat* or comprehensive or intermediate or extended or follow-up or postdischarge or post-discharge or posthospital* or post-hospital* or home or home-based or domiciliary or long-term or outpatient or ambulatory) adj3 (care? or service? or intervention?)).tw.

3	((interdisciplinary or multidisciplinary or interprofessional) adj3 (program* or management)).tw.

4	((patient adj2 (discharge or follow-up)) or (discharge? adj2 plan*) or (case adj2 management) or aftercare* or after care).tw.

5	case management/ or disease management/ or (manage* adj2 (care? or case? or disease?)).tw.

6	ambulatory monitoring/ or telemedicine/ or telenursing/ or teleconsultation/ or mobile applications/ or internet/

7	(((ambulatory or remote or distan* or outpatient) adj3 monitor*) or (telemed* or telemed* or telehealth* or tele-health* or tele-consult* or eHealth or mHealth or Mobile Health or telecare* or telenurs* or telemonitor* or teleconsult* or tele-consult* or teleconferenc* or tele-conferenc* or telecommunicat* or telecardiol* or telephon* or telemetry or phone* or messag* or consultation* or application* or mobile* or app* or software*)).tw.

8	enterostomy/ or cecostomy/ or colostomy/ or duodenostomy/ or ileostomy/ or jejunostomy/ or surgical stomas/ or ostomy/

9	(enterostom* or cecostom* or colostom* or duodenostom* or ileostom* or jejunostom* or ostom*).tw.

10	((Surgical or colon) adj2 Stoma*).tw.

11	(random* or factorial* or crossover* or cross over* or cross-over* or placebo* or (doubl* adj blind*) or (singl* adj blind*) or assign* or allocat* or volunteer*).tw.

12	crossover procedure/ or double blind procedure/ or randomized controlled trial/ or single blind procedure/

13	(animal/ or nonhuman/) not human/

14	1 or 2 or 3 or 4 or 5 or 6 or 7

15	8 or 9 or 10

16	11 or 12

17	16 not 13

18	14 and 15 and 17

Cochrane Central Register of Controlled Trials (Cochrane Library)

#1	((transition* or transfer* or continu* or coordinat* or collaborat* or seamless or integrat* or comprehensive or intermediate or extended or follow-up or postdischarge or post-discharge or posthospital* or post-hospital* or home or home-based or domiciliary or long-term or outpatient or ambulatory) near/3 (care? or service? or intervention?))

#2	(manage* near/2 (care? or case? or disease?))

#3	(interdisciplinary or multidisciplinary or interprofessional) near/3 (program* or management)

#4	patient near/2 (discharge or follow-up) or (discharge? near/2 plan*) or (case near/2 management) or aftercare* or "after care"

#5	(ambulatory or remote or distan* or outpatient) near/3 monitor*

#6	((telemed* or telemed* or telehealth* or tele-health* or tele-consult* or eHealth or mHealth or Mobile Health or telecare* or telenurs* or telemonitor* or teleconsult* or tele-consult* or teleconferenc* or tele-conferenc* or telecommunicat* or telecardiol* or telephon* or telemetry or phone* or messag* or consultation* or application* or mobile* or app* or software*)):ti,ab,kw

#7	MeSH descriptor: [Case Management] explode all trees

#8	MeSH descriptor: [Disease Management] this term only

#9	MeSH descriptor: [Continuity of Patient Care] explode all trees

#10	MeSH descriptor: [Ambulatory Care] this term only

#11	MeSH descriptor: [Long-Term Care] explode all trees

#12	MeSH descriptor: [Home Care Services] explode all trees

#13	MeSH descriptor: [Monitoring, Ambulatory] explode all trees

#14	MeSH descriptor: [Telemedicine] explode all trees

#15	MeSH descriptor: [Telenursing] explode all trees

#16	MeSH descriptor: [Mobile Applications] explode all trees

#17	MeSH descriptor: [Internet] explode all trees

#18	#1 or #2 or #3 or #4 or #5 or #6 or #7 or #8 or #9 or #10 or #11 or #12 or #13 or #14 or #15 or #16 or #17

#19	MeSH descriptor: [Enterostomy] explode all trees

#20	MeSH descriptor: [Surgical Stomas] explode all trees

#21	MeSH descriptor: [Ostomy] this term only

#22	(enterostom* OR cecostom* OR colostom* OR duodenostom* OR ileostom* OR jejunostom* OR ostom*)

#23	((Surgical or colon) near/2 Stoma*)

#24	#19 or #20 or #21 or #22 or #23

#25	#18 and #24 in Trials

CINAHL (EBSCO)

S1	MH (Case Management or Disease Management)

S2 TX (manage* N2 (care? OR case? OR disease?))

S3 MH (Continuity of Patient Care+ OR After Care OR Patient Discharge+ OR Transitional Care OR Ambulatory Care OR Long Term Care OR Home Health Care OR home health nursing) Expanders - Apply equivalent subjects Search modes - Proximity Interface - EBSCOhost Research Databases Search Screen - Advanced Search Database - CINAHL Plus with Full Text

S4 TX ((transition* OR transfer* OR continu* OR coordinat* OR collaborat* OR seamless OR integrat* OR comprehensive OR intermediate OR extended OR follow-up OR postdischarge OR Expanders - Apply equivalent subjects Search modes - Proximity Interface - EBSCOhost Research Databases Search Screen - Advanced Search Database - CINAHL Plus with Full Text 506,842 post-discharge OR posthospital* OR posthospital* OR home OR home-based OR domiciliary OR long-term OR outpatient OR ambulatory) N3 (care? OR service? OR intervention?))

S5 TX ((interdisciplinary OR multidisciplinary or interprofessional) N3 (program* OR management))

S6 TX (patient N2 (discharge OR follow-up) OR (discharge? N2 plan*) OR (case N2 management) OR aftercare* OR “after care”)

S7 MH (elecommunications+ or mobile applications)

S8 TX ((ambulatory OR remote OR distan* OR outpatient) N3 monitor* OR (telemed* OR tele-med* OR telehealth* OR tele-health* OR tele-consult* OR eHealth OR mHealth OR “Mobile Health” OR telecare* OR telenurs* OR telemonitor* OR teleconsult* OR tele-consult* OR teleconferenc* OR tele-conferenc* OR telecommunicat* OR telecardiol* OR telephon* OR telemetry or phone* OR messag* or consultation* or application* or mobile* or app* or software*))

S9 MH (Enterostomy+ or surgical stoma or ostomy)

S10 TX (enterostom* OR cecostom* OR colostom* OR duodenostom* OR ileostom* OR jejunostom* OR ostom*)

S11 TX (((Surgical or colon) N2 Stoma*)))

S12 PT (randomized controlled trial or clinical trial)

S13 TI (randomis* or randomiz* or randomly)

S14 AB (randomis* or randomiz* or randomly)

S15 MH (Clinical Trials+ or Random Assignment)

S16 S1 OR S2 OR S3 OR S4 OR S5 OR S6 OR S7 OR S8

S17 S9 OR S10 OR S11

S18 S12 OR S13 OR S14 OR S15

S19 S16 AND S17 AND S18

**Supplementary Table 1.** Reason for exclusion of studies screened at full text

| **No.** | **Study** | **Reason for exclusion** |
| --- | --- | --- |
| 1 | Aktas et al, 2024[1] | Unsuitable population |
| 2 | Alenezi et al, 2016[2] | Unsuitable intervention |
| 3 | Augestad et al, 2020[3] | Unsuitable comparator |
| 4 | Bagnasco et al, 2017[4] | Unsuitable study design |
| 5 | Beaver et al, 2010[5] | Unsuitable study design |
| 6 | Bednarski et al, 2018[6] | Unsuitable study design |
| 7 | Bohnenkamp et al, 2004[7] | Unsuitable study design |
| 8 | Boutry et al, 2021[8] | Unsuitable study design |
| 9 | Cavalli-Bjorkman et al, 2011[9] | Unsuitable study design |
| 10 | Cengiz et al, 2020[10] | Unsuitable study design |
| 11 | Chaudhri et al, 2005[11] | Unsuitable intervention |
| 12 | Cheung et al, 2003[12] | Unsuitable intervention |
| 13 | Chew et al, 2003[13] | Unsuitable study design |
| 14 | Chrobak-Bien et al, 2023[14] | Unsuitable intervention |
| 15 | Coca et al, 2015[15] | Unsuitable study design |
| 16 | Crawford et al, 2012[16] | Outcomes of interest not available |
| 17 | de Ponthaud et al, 2023[17] | Unsuitable study design (study protocol) |
| 18 | Denost et al, 2023[18] | Unsuitable study design (study protocol) |
| 19 | DuHamel et al, 2016[19] | Unsuitable population |
| 20 | Dulskas et al, 2021[20] | Unsuitable intervention |
| 21 | Dumont et al, 2013[21] | Unsuitable intervention |
| 22 | Duque et al, 2023[22] | Unsuitable study design |
| 23 | Eom et al, 2021[23] | Unsuitable population |
| 24 | Ercolano et al, 2023[24] | Unsuitable study design |
| 25 | Farroni et al, 2007[25] | Unsuitable intervention |
| 26 | Feng et al, 2022[26] | Retracted publication |
| 27 | Feng et al, 2022[27] | Unsuitable study design |
| 28 | Gao et al, 2022[28] | Retracted publication |
| 29 | Golpazir-Sorkheh et al, 2022[29] | Unsuitable study design |
| 30 | Grahn et al, 2019[30] | Unsuitable intervention |
| 31 | Grant et al, 2011[31] | Unsuitable study design |
| 32 | Grant et al, 2022[32] | Unsuitable study design |
| 33 | Hao et al, 2023[33] | Unsuitable study design |
| 34 | Heidari-Beni et al, 2022[34] | Unsuitable population |
| 35 | Hornbrook et al, 2018[35] | Unsuitable study design |
| 36 | Huang et al, 2021[36] | Unsuitable study design |
| 37 | Hubbard et al, 2020[37] | Unsuitable study design (study protocol) |
| 38 | Hubbardet et al, 2017[38] | Unsuitable study design |
| 39 | Hubbardet et al, 2019[39] | Unsuitable study design (study protocol) |
| 40 | Hyunjung et al, 2023[40] | Unsuitable intervention |
| 41 | Iovino et al, 2024[41] | Unsuitable study design (study protocol) |
| 42 | Jimenez et al, 2010[42] | Unsuitable study design |
| 43 | Jin et al, 2022[43] | Unsuitable population |
| 44 | Jinjian et al, 2011[44] | Unsuitable intervention |
| 45 | Kang et al, 2020[45] | Unsuitable intervention |
| 46 | Karadag et al, 2005[46] | Unsuitable intervention |
| 47 | Khalilzadeh et al, 2019[47] | Unsuitable population |
| 48 | Khwanhathai et al, 2023[48] | Unsuitable study design |
| 49 | Ko et al, 2023[49] | Unsuitable comparator |
| 50 | Koc et al, 2023[50] | Unsuitable intervention |
| 51 | Krouse et al, 2006[51] | Unsuitable study design |
| 52 | Krouse et al, 2015[52] | Unsuitable study design |
| 53 | Krouse et al, 2016[53] | Unsuitable study design |
| 54 | Krouse et al, 2024[54] | Unsuitable population |
| 55 | Li et al, 2024[55] | Unsuitable intervention |
| 56 | Li et al, 2024[56] | Unsuitable study design |
| 57 | Lim et al, 2015[57] | Unsuitable study design (study protocol) |
| 58 | Lim et al, 2019[58] | Unsuitable population |
| 59 | Lingying et al, 2023[59] | Unsuitable study design |
| 60 | Liu et al, 2019[60] | Unsuitable study design |
| 61 | Mazanec et al, 2021[61] | Unsuitable population |
| 62 | Michelone et al, 2004[62] | Unsuitable study design |
| 63 | Mo et al, 2020[63] | Unsuitable study design |
| 64 | Momeni et al, 2023[64] | Unsuitable study design |
| 65 | Munro et al, 2023[65] | Unsuitable study design |
| 66 | NasiriZiba et al, 2020[66] | Unsuitable study design |
| 67 | Nasvall et al, 2017[67] | Unsuitable study design |
| 68 | Nehemiah et al, 2023[68] | Unsuitable population |
| 69 | Orsini et al, 2013[69] | Unsuitable study design |
| 70 | Otto et al, 2008[70] | Unsuitable intervention |
| 71 | Pan et al, 2022[71] | Retracted publication |
| 72 | Pate et al, 2020[72] | Unsuitable study design |
| 73 | Pittman et al, 2022[73] | Unsuitable study design |
| 74 | Pouresmail et al, 2019[74] | Unsuitable intervention |
| 75 | Pozzar et al, 2024[75] | Unsuitable population |
| 76 | Quan et al, 2024[76] | Unsuitable intervention |
| 77 | Rahimi et al, 2021[77] | Unsuitable population |
| 78 | Rock et al, 2022[78] | Unsuitable population |
| 79 | Rojanasarot et al, 2018[79] | Unsuitable study design |
| 80 | Santos et al, 2016[80] | Unsuitable study design |
| 81 | Sasaki et al, 2021[81] | Unsuitable study design |
| 82 | Seo et al, 2019[82] | Unsuitable intervention |
| 83 | Silva et al, 2003[83] | Unsuitable study design |
| 84 | Souza Freitas et al, 2023[84] | Outcomes of interest not available |
| 85 | Sun et al, 2014[85] | Unsuitable study design |
| 86 | Sun et al, 2016[86] | Unsuitable study design |
| 87 | Sun et al, 2018[87] | Unsuitable study design (study protocol) |
| 88 | Taylan et al, 2019[88] | Unsuitable study design |
| 89 | Van der Storm et al, 2024[89] | Unsuitable intervention |
| 90 | Van et al, 2022[90] | Unsuitable study design (study protocol) |
| 91 | Van et al, 2023[91] | Unsuitable study design (study protocol) |
| 92 | Wan et al, 2021[92] | Unsuitable study design (study protocol) |
| 93 | Wang et al, 2018[93] | Unsuitable population |
| 94 | Wang et al, 2023[94] | Unsuitable population |
| 95 | Weinstein et al, 2021[95] | Unsuitable study design |
| 96 | Werther et al, 2024[96] | Unsuitable study design |
| 97 | Xu et al, 2023[97] | Unsuitable population |
| 98 | Xu et al, 2023[98] | Unsuitable study design |
| 99 | Yan et al, 2023[99] | Unsuitable study design |
| 100 | Yang et al, 2020[100] | Unsuitable population |
| 101 | Ye et al, 2019[101] | Unsuitable population |
| 102 | Yu et al, 2021[102] | Outcomes of interest not available |
| 103 | Zhang et al, 2021[103] | Unsuitable population |
| 104 | Lo et al. 2010 [104] | Unsuitable intervention |
| 105 | Lo et al. 2011 [105] | Unsuitable intervention |
| 106 | Oner Cengiz et al. 2026 [106] | Unsuitable intervention |
| 107 | Rajabi et al. 2025 [107] | Unsuitable intervention |
| 108 | Fang et al. 2025 [108] | Unsuitable study design |

**Supplementary Table 2.** Characteristics and quality appraisal of the included studies (n = 18).

| **Study (country)** | **Sample size^a^** | **Participants^a^** | **Intervention group** | **Control group** | **Outcomes: tools/assessment point** |
| --- | --- | --- | --- | --- | --- |
| Geng, W, 2019  (China) | Single-center  N=60 (Adults only)  **Randomized**: 30 / 30 (Adults)  **Analyzed**: 30 / 30 (Adults) | **Mean age (SD), yr**: 45.4 (13.3) vs. 45.4 (13.3)  **Female, n (%)**: 13 (43.3) vs. 12 (40.0); Total, n (%): 25 (41.7)  **Diagnosis (%)**: Not specified  **Stoma type (%)**: Ileostomy 14 (46.7) vs. 16 (53.3); Colostomy 16 (53.3) vs. 14 (46.7)  **Stoma status (%)**: Not specified | **Intervention category**: Technology-Mediated Post-Discharge Support  **Theoretical framework:** Not specified  **Recipient:** Patients and their families  **Content**: The continuous nursing team developed a 6-month continuing care program. This included postoperative stoma care training for families, regular hospital reviews, weekly phone follow-ups, and organized social activities. A WeChat group was established for real-time support, and psychological counseling was provided to build patient confidence.  **Delivery personnel**: Nurse-led (Specialist nurses)  **Environment:** Hospital and home  **Communication method**: Telephone, WeChat, face-to-face  **Initiation of intervention**: After surgery  **Frequency**: Weekly phone calls, regular WeChat interaction.  **Duration**: 6 m | **Routine nursing care**: Monthly routine telephone follow-ups after discharge. | **Complications**:  Peripheral dermatitis, invagination, stenosis, and para hernia  Assessed via follow-up  / within 6 months of discharge  **QoL**: Self-developed scale  **Anxiety**: SAS  **Depression**: SDS  **Satisfaction**  / 6 months after discharge |
| Hu H, 2020 (China) | Single-center N=108 **Randomized**: 54 / 54 **Analyzed**: 54 / 54 | **Mean age (SD), yr**: 54.2 (7.0) vs. 53.8 (6.8) **Female, n (%)**: 24 (44.4) vs. 25 (46.3); Total (%): 49 (45.4) **Diagnosis (%)**: Colon Cancer 20 (37.0) vs. 22 (40.8); Rectal cancer 34 (63.0) vs. 32 (59.3) **Stoma type (%)**: Colostomy (100) **Stoma status (%)**: Temporary (100) | **Intervention category**: Technology-Mediated Post-Discharge Support **Theoretical framework**: Not specified **Recipient**: Patient and family members **Content**: The nursing plan established 3 days before discharge, including telephone follow-up (72 hours after discharge and once a week to assess complications, psychological wellbeing, medication adherence, etc.), WeChat follow-up (regular sharing of disease knowledge, skill videos) and outpatient follow-up once a month. In addition, face-to-face communication between nurses and patients were conducted monthly. **Delivery personnel**: MDT (4 nurses, 1 physician, and 1 stomatologist) **Communication method**: Telephone, WeChat, outpatient, face-to-face  **Environment**: Hospital and home **Initiation of intervention**: 72h after discharge (Plan made before discharge; active follow-up started post-discharge) **Frequency**: Weekly phone calls, regular WeChat interaction, monthly outpatient visits and group chats. **Duration**: 3m after discharge | **Routine nursing**: In-hospital education, and monthly telephone follow-up after discharge. | **Complications**:  Bleeding, peristomy infection/stenosis/dermatitis  Diagnosed by medical staff  / 3m after discharge  **QoL**: SF-36  **Self-care**: ESCA  **Anxiety:** SAS  **Depression**: SDS  / 1 day post-surgery and 3m post-discharge  **Satisfaction**: Theself-designed questionnaire  / 3m after discharge |
| Li SQ, 2024 (China) | Single-center N=92 **Randomized**: 46 / 46 **Analyzed**: 41 / 41 | **Mean age (SD), yr**: 62.5 (10.6) vs. 55.5 (15.3) **Female, n (%)**: 19 (46.3) vs. 22 (53.7); Total, n (%): 41 (50.0) **Diagnosis (%)**: CRC (100) **Stoma type, n (%)**: Ileostomy 21 (51.2) vs. 25 (61.0); Colostomy 20 (48.8) vs. 16 (39.0) **Stoma status, n (%)**: Temporary 37 (90.2) vs. 34 (82.9); Permanent 4 (9.8) vs. 7 (17.1) | **Intervention category**: Comprehensive Perioperative Support **Theoretical framework**: Orem’s self-care model **Recipient**: Patients and their families **Content**: Preoperatively, patients received education on disease-related knowledge and stoma care, along with psychological support. Postoperatively, they were guided to assess stoma mucosa color and peristomal skin condition. Researchers provided the stoma pouch replacement, cleaning, and emptying procedure for patients and their families. Before discharge, both patients and their families were guided in stoma self-care and complication recognition. After discharge, regular telephone follow-ups or return visits were conducted to evaluate the patient’s self-care ability. **Delivery personnel**: Nurse-led **Communication method**: Face-to-face, telephone or return visit  **Environment**: Hospital and home **Initiation of intervention**: Before surgery **Frequency**: Continuous support during hospitalization (1-2 days before surgery, day, 1-2, 3-4, 5-8 days after surgery); follow up at 1m and 3m after discharge. **Duration**: 3m after discharge | **Routine care**: Admission assessment, preoperative care, stoma care, and regular follow-up at 1m and 3m post-discharge. | **QoL**: Stoma QoL scale **Self-care**: Ostomy self-care ability scale  / at discharge, 1m and 3m after discharge **LOS**: Medical records  / at discharge **Unplanned readmission**: Medical records  / not specified |
| Lin L, 2024 (China) | Single-center N=160 I/C **Randomized**: 80 / 80 I/C **Analyzed**: 78 / 75 | **Mean age (SD), yr**: 61.7 (11.9) vs. 60.4 (12.1) **Female, n (%)**: 23 (29.5) vs. 24 (32.0); Total, n (%): 43 (30.7) **Diagnosis, n (%)**: Colon cancer: 5 (6.4) vs. 4 (5.3); Rectal cancer: 73 (93.6) vs. 71 (94.7) **Stoma type, n (%)**: Ileostomy: 75 (96.2) vs. 73 (97.3); Colostomy: 3 (3.9) vs. 2 (2.7) **Stoma status**: Not specified | **Intervention category**: Comprehensive Perioperative Support **Theoretical framework**: Not specified **Recipient**: Patient and family caregiver **Content**: The nurse-led discharge planning program comprised four phased interventions: Stage 1 (Admission): Comprehensive screening and personalized care plan formulation. Stage 2 (24h post admission): Inpatient stoma care training, psychosocial support, and health education for transition from hospital to home. Stage 3 (24h pre-discharge): Medication reconciliation, discharge checklist completion, and referral coordination. Stage 4 (90d post-discharge): Structured follow-up via telephone, outpatient visits, and WeChat support. Delivery personnel: MDT (Surgeons, nutritionists, psychotherapists, nurse managers, nurse enterostomists, and primary nurses) **Communication method**: Face-to-face, telephone, WeChat, outpatient visit **Environment**: Hospital, home, and clinic **Initiation of intervention**: At admission (The day the patient was admitted) **Frequency**: Continuous during hospitalization; telephone follow-ups at 7, 14, 30, and 90 days post-discharge; outpatient visit at 4 weeks. **Duration**: 3m after discharge | **Usual care**: Usual admission education, standard surgical care, telephone (1 week) and outpatient (1 month) follow-up after discharge. | **Complications**:  Stoma complications (yes / no)  Medical records / at discharge, 1m, 3m after discharge **QoL**: Stoma QoL Scale  **Self-efficacy**: SSES / baseline, at discharge, 1m and 3m after discharge **LOS**: Medical records  / at discharge **Unplanned readmission**: Medical records  / 1m, 3m after discharge |
| Lin LY, 2025 (China) | Single-center N=130 I/C **Randomized**: 65 / 65 I/C **Analyzed**: 62 / 62 | **Mean age (SD), yr**: 62.7 (10.2) vs. 61.6 (11.9) **Female, n (%)**: 18 (29.0) vs. 26 (41.9); Total, n (%): 44 (35.5) **Diagnosis (%)**: CRC (100) **Stoma type, n (%)**: Ileostomy 58 (93.5) vs. 56 (90.3); Colostomy 4 (6.5) vs. 6 (9.7) **Stoma status, n (%)**: Temporary (100) | **Intervention category**: Comprehensive Perioperative Support **Theoretical framework**: Skinner’s teaching theory **Recipient**: Patients and their families **Content**: Preoperatively, the researchers assisted patients and their families in becoming familiar with the stoma and its impact on daily life. Postoperatively, patients were guided to learn the replacement of stoma pouches, identify and prevent common complications, and complete stoma care independently. After discharge, researchers provided compensatory guidance to patients and families through follow up. **Delivery personnel**: Nurse-led (Well-qualified primary care nurses and ET) **Communication method**: Face-to-face and telephone **Environment**: Hospital and home **Initiation of intervention**: Before surgery **Frequency**: Continuous support during hospitalization (Before surgery, 1, 2-3, 3-5, 5-7 days after surgery, and the days of discharge); follow up after discharge. **Duration**: 3m after discharge | **Routine care**: Discharge instructions, standard admission education, surgical care, telephone (1 week) and outpatient (1 m) after discharge | **Complication**:  Stoma complications (yes / no)  Assessed via follow-up  / on the day of discharge, 1m and 3m after discharge |
| Liu, Y, 2021 (China) | Single-center N=126 I/C **Randomized**: 63 / 63 I/C **Analyzed**: 63 / 63 | **Mean age (SD), yr**: 43.7 (4.1) vs. 44.6 (4.3) **Female, n (%)**: 30 (47.6) vs. 28 (44.4); Total, n (%): 58 (46.0) **Diagnosis (%)**: Colorectal cancer (CRC, 100) **Stoma type (%)**: Colostomy (100) **Stoma status (%)**: Permanent (100) | **Intervention category**: Technology-Mediated Post-Discharge Support **Theoretical framework**: Not specified **Recipient**: Patients alone **Content**: Health knowledge and self-care skills were delivered via WeChat/QQ platforms using pictures, short videos, text and other forms. This included ostomy appliance techniques, dietary guidance, exercise guidance, etc. **Delivery personnel**: Nurse-led (Responsible nurse) **Communication method**: WeChat or QQ **Environment**: Home  **Initiation of intervention**: 1-3d before discharge **Frequency**: Regular pushes of information (Specific frequency not stated). **Duration**: Not specified | **Routine care**: Standard health education, stoma care, and regular telephone follow-up after discharge. | **Complications**:  Parastomal hernia, fecal dermatitis, stricture of stoma, intestinal obstruction, and stoma retraction  Medical records / after intervention (timepoint not specified) **Qo**L: WHOQOL-BREF  **Anxiety**: HAMA **Depressio**n: HAMD / baseline and after intervention (timepoint not specified) |
| Ozel H, 2025  (Türkey: UMIC) | Single-center  N=70  I/C: Randomized 35 / 35  I/C: Analyzed 35 / 35 | **Mean age (SD), yr:** 55.8 (13.1) vs. 59.8 (9.1)  **Female, n (%):** 12 (34.3) vs. 14 (40.0); Total: 26 (37.1)  **Diagnosis (%): CRC (77.2 vs 80.0)**  **Stoma type, n (%):** Ileostomy 22 (62.9) vs. 16 (45.7); Colostomy 13 (37.1) vs. 19 (54.3)  **Stoma status (%):** Not specified | **Intervention category:** Technology-Mediated Post-Discharge Support  **Theoretical framework:** Not specified  **Recipient:** Patients and their families  **Content:** Participants received access to the "web-based education program" between the 3rd and 7th postoperative days (via a card including the website address, QR code, and their username). After discharge, they and their families could repeatedly watch the educational content and patients could ask questions or send a picture/video through the "Ask a Nurse" textbox on the website. Researchers tracked participants' access and usage through the website's administrator panel.  **Delivery personnel:** Nurse-led  **Communication method:** Web-based program (website with videos, text, and "Ask a Nurse" feature for questions)  **Environment:** Hospital and home (or anywhere with internet access)  **Initiation of intervention:** Between 3rd and 7th day postoperatively  **Frequency:** Self-paced, could access program content as many times as desired  **Duration**: 30 days after surgery | **Usual care:** Disease information and ostomy care were provided by physicians or nursing staff before and after surgery (on postoperative days 1 and 3). | **Complications:** OCAF  Leakage, peristomal skin complications, pain and bleeding  / 1m after discharge  **QoL:** CoH-QoL-OQ  / at baseline and 1m after discharge |
| Özkaya E, 2024 (Turkey) | Single-center N=60  I/C **Randomized**: 30 / 30 I/C **Analyzed**: 30 / 30 | **Mean age (SD), yr**: 58.5 (13.5) vs. 64.2 (10.2) **Female, n (%)**: 9 (30.0) vs. 6 (20.0); Total, n (%): 15 (25.0) **Diagnosis, n (%)**: CRC 24 (80.0) vs. 24 (80.0) Total, n (%): 48 (80.0) **Stoma type (%)**: Colostomy 13 (43.3) vs. 12 (40.0); Ileostomy 17 (56.7) vs. 18 (60.0) **Stoma status (%)**: Temporary 18 (60.0) vs. 19 (63.3); Permanent 12 (40.0) vs. 11 (36.7) | **Intervention category**: Technology-Mediated Post-Discharge Support **Theoretical framework**: Not specified **Recipient**: Patients alone **Content**: Participants received an educational booklet ("Life with Ileostomy/Colostomy"). After discharge, they received four individual education sessions via videoconference. The content included stoma materials, skin care, bag changing on a model, diet, medication, physical activity, and social life. **Delivery personnel**: Nurse-led (Experience in stoma care for 2 years) Communication method: Video conferencing (Zoom, WhatsApp), telephone **Environment**: Home **Initiation of intervention**: 5d after discharge **Frequency**: Four sessions at 5 days, 10 days, 15 days, and 2 months after discharge. Each session lasted ~40 minutes **Duration**: 2m after discharge | **Routine care**: In-hospital education and telephone follow-up at 2m post-discharge. | **Complications**:  Hernia, skin irritation, retraction, mucocutaneous separation, and prolapse  Visual assessment by researcher / 2m after discharge **Self-efficacy**: SSES **Stoma Adaptation**: OAI-23 / baseline (before discharge) and 2m after discharge **Unplanned readmission**: Patient self-report  / 2 months after discharge |
| Su X, 2021 (China) | Multi-center (4)  N=124 I/C **Randomized**: 60 / 64 I/C **Analyzed**: 50 / 57 | **Mean age (SD), yr**: 57.0 (14.7) vs. 59.1 (12.9) **Female, n (%)**: 15 (30.0) vs. 25 (43.9); Total, n (%): 40 (37.4) **Diagnosis (%)**: Rectal cancer (100) **Stoma type, n (%)**: Ileostomy 41 (82.0) vs. 40 (70.2); Colostomy 9 (18.0) vs. 17 (29.8) **Stoma status (%)**: Temporary (100) | **Intervention category**: Comprehensive Perioperative Support **Theoretical framework**: Bandura’s self-efficacy theory  **Recipient**: Patients alone **Content**: Evidence-based continuing care bundle. Participants received the self-management manual at admission and learned stoma care skills post-surgery. Structured telephone follow-up was conducted to assess participants' emotions, symptoms, and self-efficacy, and an outpatient visit at 4 weeks after surgery was conducted to evaluate stoma-related complications. **Delivery personnel**: Enterostomal therapist (ET) or Wound, Ostomy, and Continence Nurse (WOCN) **Communication method**: Telephone, Face-to-Face outpatient visit **Environment**: Hospital and home **Initiation of intervention**: Within 24 h of admission  **Frequency**: Continuous guidance during hospitalization; 4 telephone follow-ups (3–7 days, 14–20 days, 27-30 days, 87–90 days after discharge) and 1 outpatient visit post-discharge. **Duration**: 3m after discharge | **Usual care**: Routine health education, stoma care, and telephone or outpatient follow-up as needed. | **Complications**:  Dermatitis, mucocutaneous separation, stoma prolapse, stoma stenosis, parastomal incision infection, stoma retraction, parastomal hernia and granuloma  Diagnosed by ET or WOCN based on a preset checklist  / at discharge, 4w and 12w after surgery **QoL**: Stoma QOL scale  **Self-efficacy**: SSES  / at discharge, 4w and 12w after surgery **Satisfaction**: Face-to-face questionnaires  / 12w after surgery |
| Wang J, 2026  (China: LMIC) | Single-center  N=200  I/C: **Randomized:** 100 / 100  I/C: **Analyzed:** 100 / 100 | **Age, n (%), yr:**  30-50: 10 (10.0) vs. 10 (10.0);  51-70: 57 (57.0) vs. 58 (58.0);  ≥71: 33 (33.0) vs. 32 (32.0)  **Female, n (%):** 46 (46.0) vs. 51 (51.0); Total: 97 (48.5)  **Diagnosis:** Colorectal cancer (CRC) (100%)  **Stoma type**: Not specified  **Stoma status:** Permanent (100%) | **Intervention category:** Comprehensive Perioperative Support  **Theoretical framework:** Transtheoretical Model (TTM)  **Recipient:** Patients alone  **Content:** Stage-tailored program using 5 innovative tools: enterostomy education skirt (pre-intention stage), 3D interactive nursing teaching app (intention stage), VR training simulation system (preparation stage), complication demonstration models simulating 36 complications (action stage), and multi-channel support (24h hotline, ostomy workshops and popular science lectures) (maintenance stage).  **Delivery personnel:** Nurse-led  **Communication method:** Face-to-face, mobile app, VR, telephone, online platform, workshops, and popular science lectures  **Environment:** Hospital and home  **Initiation of intervention:** Preoperative  **Frequency:** Self-paced app/VR; monthly workshops and popular science lectures  **Duration:** 1m after discharge | **Usual care:** Routine education, preoperative site marking, dietary guidance, weekly video, predischarge training, WeChat official account, telephone follow-up (1 week, then monthly), outpatient follow-up at 1 month. | **Complications:** SAS  Peristomal skin problems, stenosis, prolapse, parastomal hernia, retraction, and mucocutaneous separation  Assessed by the international ET  / 1m after discharge  **Self-care ability:** ASAS-R-C scale  / at baseline and one day before discharge  **Satisfaction:** Self-designed questionnaire  / 1m after discharge |
| Wang Y, 2024 (China) | Single-center N=120 I/C **Randomized**: 60 / 60 I/C **Analyzed**: 60 / 60 | **Mean age (SD), yr**: 51.3 (11.5) vs. 50.4 (11.4) **Female, n (%)**: 23 (38.3) vs. 25 (41.7); Total, n (%): 48 (40.0) **Diagnosis**: Not specified **Stoma type (%)**: Colostomy (100) **Stoma status (%)**: **Permanent** (100) | **Intervention category**: Technology-Mediated Post-Discharge Support ET-led visual health education combined with peer education **Theoretical framework**: Not specified **Recipient**: Patients alone **Content**: A MDT conducted intensive training for peer educators 3 sessions per week for 4 weeks (2h/session), covering stoma care and psychological supports. Peer educators and patients were then divided into six groups at a 2:10 ratio. The participants received personalized guidance once a week (via telephone/WeChat/home visits) and participated in group discussions once a month. **Delivery personnel**: Multidisciplinary team (ET-led, nurses, psychologist, nutritionist, and peer educators) **Environment**: After discharge **Communication method**: Telephone, WeChat, face-to-face (Home visits, group discussions) **Initiation of intervention**: After discharge **Frequency**: Weekly peer consultations and monthly group discussions. **Duration**: 3 m after discharge | **Routine care**: Pre-discharge instructions and standard postoperative follow-up. | **Complications:**  Stoma bleeding, skin and mucosal separation, stoma edema, stoma prolapse, and dermatitis around the stoma  / 3 months after surgery **Qo**L: Stoma QoL scale  **Self-care**: ESCA  **Stoma Adaptation**: OAS / baseline (0 day), 1m, and 3m after discharge **Satisfaction**: Self-made questionnaires  / 3m after discharge |
| Wen SL, 2019 (China) | Multi-center (3) N= 92 I/C **Randomized**: 47 / 45 I/C **Analyzed**: 47 / 45 | **Age, n (%), yr:** 52.8 ± 11.13 <45 14 (29.8) vs. 13 (28.9);  46-55 13 (27.7) vs. 16 (35.6);  56-65 13 (27.7) vs. 7 (15.6);  >66 7 (14.9) vs. 9 (20.0) **Female, n (%)**: 17 (36.2) vs. 20 (44.4); Total (%): 37 (40.2) **Diagnosis (%)**: CRC (100) **Stoma type (%)**: Colostomy (100) **Stoma status (%)**: Permanent (100%) | **Intervention category**: Comprehensive Perioperative Support **Theoretical framework**: Transtheoretical model  **Recipient**: Patients alone **Content**: Through four structured face-to-face sessions, researchers provided targeted supports to patients based on the stages of change (precontemplation, contemplation, preparation, and maintenance). The sessions covered needs assessment, goal setting, stoma care training, and psychosocial adaptation guidance. **Delivery personnel**: Nurse-led (1 researcher, 1 ET nurse) **Communication method**: Face-to-face **Environment**: Hospital  **Initiation of intervention**: After admission  **Frequency**: Four sessions in total (At baseline, before discharge, 1m, and 3m post-discharge). Each session lasted about 1 hour. **Duration**: 6m after discharge | **Conventional care**: General post-operative health education, and telephone follow-up post-discharge. | **Self-efficacy**: Self‐efficacy subscale / baseline, 2 days before discharge, 1m, 3m, and 6m after discharge |
| Xia L, 2020 (China) | Single-center N=155 I/C **Randomized**: 81 / 74 I/C **Analyzed**: 81 / 74 | **Age, n (%), yr**:  <50: 42 (51.9) vs. 36 (48.6) >50: 39 (48.1) vs. 38 (51.4) **Female, n (%)**: 37 (45.7) vs. 35 (47.3); Total (%): 72 (46.5) **Diagnosis (%)**: Rectal cancer (100) **Stoma type (%)**: Colostomy (100) **Stoma status (%)**: Permanent (100) | **Intervention category**: Comprehensive Perioperative Support **Theoretical framework**: Not specified **Recipient**: Patient and family caregiver **Content**: Researchers provided stoma care education and self-management manual for patients. Post-discharge, colostomy therapist made home visits and real-time consultations through online platforms (WeChat, QQ, and telephone). Meanwhile, a designated family caregiver was trained to assist with daily care, monitor the patient’s condition, and communicate regularly with the colostomy therapist. **Delivery personnel**: Colostomy therapist  **Communication method**: WeChat, blog, QQ, telephone, Face-to-Face **Environment**: Hospital and home **Initiation of intervention**: Within 24h of admission **Frequency**: Continuous support during hospitalization; home visits and online/phone contact as needed post-discharge. **Duration**: 3m after discharge | **Routine Care**: Standard stoma care, and health education before discharge. | **Complications**:  Narrow, prolapse, hernia, retraction, separation of skin from mucous membrane  Diagnosed by ET (recorded as Mean ± SD of events)  / at discharge, 1m and 3m after discharge  **QoL**: Stoma QOL scale **Self-efficacy**: SSES  / at discharge, 1m and 3m after discharge **Anxiety**: STAI  / before surgery and 3m after discharge **Satisfaction**: Degree of satisfaction table / 3m after discharge |
| Xu S, 2018 (China) | Single-center N=48 I/C **Randomized**: 20 / 28 I/C **Analyzed**: 20 / 28 | **Mean age (SD), yr**: 60.9 (11.5) vs. 61.1 (13.6) **Female, n (%)**: 6 (30) vs. 9 (32.1); Total, n (%): 15 (31.3) **Diagnosis (%)**: CRC (100) **Stoma type (%)**: Colostomy (100) **Stoma status (%)**: Permanent (100) | **Intervention category**: Comprehensive Perioperative Support **Theoretical framework**: Self-Efficacy Theory (Bandura) Recipient: Patients alone **Content**: Participants received a 3m self-efficacy intervention, including direct and vicarious experiences and psychological support in the first month and visits or calls in the latter two months. **Delivery personnel**: Nurse-led **Communication method**: Face-to-face sessions, telephone calls or home visits **Environment**: Hospital and home **Initiation of intervention**: 1 week post-operation **Frequency**: During hospitalization: 4 sessions (1/week for the first month); Month 2: 2 calls/visits; Month 3: 1 call/visit. **Duration**: 3m post-surgery | **Routine nursing care**: Not specified | **QoL**: EORTC QLQ-C30 / 3m after discharge **Self-efficacy**: SSES / 10 days, 1m, and 3m after discharge |
| Yilmazer T, 2020 (Turkey) | Single-center N=60 I/C **Randomized**: 30 / 30 I/C **Analyzed**: 30 / 30 | **Mean age (SD), yr**: 54.9 (9.9) vs. 59.1 (10.4) **Female, n (%)**: 16 (53.3) vs. 16 (53.3); Total, n (%): 32 (53.3) **Diagnosis (%)**: Not specified **Stoma type, n (%)**: Ileostomy 4 (13.3) vs. 6 (20.0); Colostomy 26 (86.7) vs. 24 (80.0) **Stoma status, n (%)**: Temporary 23 (76.7) vs. 18 (60.0); Permanent 7 (23.3) vs. 12 (40.0) | **Intervention category**: Comprehensive Perioperative Support **Theoretical framework**: Information-Motivation-Behavioral Skills model **Recipient**: Patients alone **Conten**t: Post-surgery, participants received the Handbook of Living with an Ostomy and 40-minute training delivered by researchers through lecture, demonstration, and discussion. Researchers provided positive behavioral feedback, postoperative ostomy care and counseling (telephone/face-to-face) post-discharge. **Delivery personnel**: Nurse-led (Clinical nurse) **Communication method**: Face-to-face, telephone **Environment**: Hospital and home **Initiation of intervention**: 1-2 d post-surgery **Frequency**: One in-hospital training session at 1-2d post-surgery; counseling at 1m and 3m post-discharge. **Duration**: 3m after discharge | **Routine ostomy care**: Routine ostomy care, and health consultation post-discharge. | **QoL**: SF-36 / 6m after discharge **Self-care**: BAF / 6m after discharge |
| Zhang JE, 2013 (China) | Multi-center (7) N=119 I/C **Randomized**: 59 / 60 I/C **Analyzed**: 52 / 51 | **Mean age (SD), yr**: 52.9 (13.3) vs. 55.3 (13.7) **Female, n (%)**: 21 (40.4) vs. 15 (29.4); **Total, n (%)**: 36 (35.0) **Diagnosis (%)**: Rectal cancer (100) **Stoma type (%)**: Colostomy (100) **Stoma status (%)**: Permanent (100) | **Intervention category**: Technology-Mediated Post-Discharge Support **Theoretical framework**: Bandura’s Social Learning Theory (SLT) **Recipient**: Patients alone **Content**: Certified enterostomal nurses delivered structured telephone interventions to assess patients' stoma complications, self-care abilities, and emotional status post-discharge. The phone follow-up plan was developed based on SLT, including self-care skills accomplishment, vicarious experiences, verbal persuasion, and emotional arousal.  **Delivery personnel**: Enterostomal nurse  **Communication method**: Telephone follow-up **Environment**: Home **Initiation of intervention**: 3-7d after discharge **Frequency**: Two to three calls over the first month post-discharge (3-7, 14-20 days or optional 23-27 days). **Duration**: 1m (23-27 days after discharge) | **Routine discharge care**: Health education, and outpatient follow-up. | **Complications**:  Skin irritation, stomal retraction/stenosis/necrosis, mucocutaneous separation, bleeding, prolapse, peristomal hernia, granuloma, mechanical injury, and other stoma complications  Identified by an enterostomal nurse according to a preset checklist  / at discharge, 1m and 3m after discharge **Self-efficacy**: SSES **Ostomy adjustmen**t: OAS  / at discharge, 1m and 3m after discharge  **Satisfaction**: Patient self-reported / baseline, 1m, 3m after discharge |
| Zhang X, 2020 (China) | Multi-center (3) N=119 I/C **Randomized**: 60 / 59 I/C **Analyzed**: 55 / 53 | **Mean age (SD), yr**: 58.6 (8.6) vs. 59.4 (8.0) **Female, n (%)**: 18 (30.0) vs. 20 (34.0); Total, n (%): 38 (31.9) **Diagnosis (%)**: CRC (100) **Stoma type (%)**: Colostomy (100) **Stoma status (%)**: Permanent (100) | **Intervention category**: Comprehensive Perioperative Support **Theoretical framework**: Timing It Right  **Recipient**: Patient alone **Content**: The intervention plan includes five different phases of the disease process: the diagnosis phase, the perioperative phase, the discharge preparation phase, the early discharge adjustment phase, and the late discharge adaptation phase (4-6m after discharge). Each phase of the plan covered knowledge introduction, medication guidance, life care, rehabilitation guidance, psychological guidance and diet guidance.  **Delivery personnel**: MDT (1 chief physician, 1 ostomy therapist, five nurses and one physician, completed by four nurses ) **Communication method**: Face-to-face, WeChat, outpatient and telephone  **Environment**: Hospital, home, and clinic **Initiation of intervention**: Before surgery (Disease diagnosis phase) **Frequency**: Month 1: 2 phone calls per week + 2 clinic visit; Month 2: 1 phone call per week + 2 clinic visit; Month 3: 2 phone call + 1 clinic visit every 3 weeks; Month 4: 1 phone call per month + 1 clinic visit per month (30-40 min / each intervention). **Duration**: 6m after discharge | **Routine care**: Health education, stoma care, discharge guidance, and routine telephone follow-up and outpatient visits after discharge. | **Complications**:  Peristomal irritant dermatitis, peristomal hernia, stoma prolapse, stoma stenosis, urine dermatitis, stoma bleeding, and candida infection  Enterostomal therapist diagnosis / at discharge, 3m and 6m after discharge **Qo**L: Stoma QoL scale **Self-care**: ESCA  / baseline, discharge, 3m and 6m after discharge |
| Zhou LH, 2025 (China) | Single-center N=160 I/C **Randomized**: 80 / 80 I/C **Analyzed**: 75 / 75 | **Mean age (SD), yr**: 62.7 (7.6) vs. 62.2 (8.5) **Female, n (%)**: 21 (28.0) vs. 33 (44.0); Total, n (%): 54 (36.0) **Diagnosis (%)**: CRC (100) **Stoma type, n (%)**: Ileostomy - small intestine stoma 48 (64.0) vs. 45 (60.0); Colostomy 27 (36.0) vs. 30 (40.0) **Stoma status, n (%)**: Temporary 29 (38.7) vs. 28 (37.3); Permanent 46 (61.3) vs. 12 (62.7) | **Intervention category**: Comprehensive Perioperative Support **Theoretical framework**: Not specified **Recipient**: Patients and their families **Content**: During hospitalization, researchers provided patients with stoma-related education, stoma-care process, management of stoma complications, emotional assessment and psychological support. A series of educational tools were developed and utilized for patients, including stoma care videos, graphical pouch-change procedure cards, etc. After discharge, regular telephone follow-ups were conducted to assess stoma condition and self-care ability. **Delivery personnel**: MDT (Nurses, 1 ET, 1 gastrointestinal surgeon) **Communication method**: Face-to-face and telephone  **Environment**: Hospital and home **Initiation of intervention**: At admission **Frequency**: Continuous support during hospitalization (At admission, 1-2 days after admission, preoperative day; 1 day, 2-6 days post-surgery; 1 day before discharge, and the day of discharge); phone contact at 1 day, 1 week, 1m, 3m and 6m after discharge. **Duration**: 6m after discharge | **Routine postoperative care**: Routine perioperative education, preoperative stoma care, and telephone follow-up at 1m post-discharge. | **Complications**:  Dermatitis caused by fecal material, stoma retraction, stoma edema, separation of skin and mucous membranes, stoma low flatness  Collected by telephone  / not specified **Qo**L: GIQLI **Self-care**: ESCA  / postoperative day 1, on day of discharge, and 1m after discharge |
| **Abbreviations**: MDT, Multidisciplinary team; ET, Enterostomal therapist; QoL, Quality of Life; SAS, Self-rating Anxiety Scale (anxiety); SDS, Self-rating Depression Scale; SF-36, 36-Item Short Form Survey; ESCA, Exercise of Self-Care Agency Scale; Stoma QoL, Stoma Quality of Life Scale; LOS, Length of stay; SSES, Stoma Self-efficacy Scale; WHOQOL-BREF, World Health Organization Quality of Life Instruments; HAMD, Hamilton Depression Rating Scale; HAMA, Hamilton Anxiety Rating Scale; OCAF, Ostomy Complications Assessment Form; COH-QOLOQ, City of Hope-Quality of Life-Ostomy Questionnaire; SAS, Stoma Assessment Scale (SAS); ASAS-R-C, Self-Care Ability Assessment Scale Revised Chinese; OAI-23, Ostomy Adjustment Inventory-23; OAS, Ostomy Adjustment Scale; STAI, State-Trait Anxiety Inventory; EORTC QLQ-C30, European Organization for Research and Treatment of Cancer Quality of Life Questionnaire Core 30; BAF, The Behavior Assessment Form; GIQLI, Gastrointestinal Quality of Life Index Scale.  **Notes:** ^a^ Intervention (I) versus Control (C) unless otherwise specified. | | | | | |

**Supplementary Table 3.** Intervention Classification for Continuous Care in Stoma Patients

Based on the 18 included studies, continuous care interventions were classified into two categories according to their primary delivery mode, timing, and complexity.

| Intervention type | Definition | Rationale |
| --- | --- | --- |
| Comprehensive Perioperative Support | This category includes structured, multi-component interventions that begin during the preoperative or immediate postoperative hospital stay and continue into the post-discharge period. These models are often led by a multidisciplinary team or specialist nurses and combine in-person education and skills training with planned post-discharge follow-up (which may include home visits, outpatient clinics, or remote contact). The key characteristic is the seamless integration of in-hospital preparation with post-discharge support. | These interventions are holistic and intensive. They aim to prepare the patient thoroughly before they leave the hospital and provide a robust support system to transition them from hospital to home, addressing a wide range of physical and psychosocial needs. |
| Technology-Mediated Post-Discharge Support | This category consists of interventions that are primarily delivered after the patient has been discharged from the hospital, using technology as the main mode of communication. This includes structured telephone follow-ups, videoconferencing, or the use of online platforms (like WeChat) and mobile apps. While some initial in-hospital instruction may occur, the core of the randomized intervention is the remote support provided post-discharge. | These interventions focus on bridging the care gap after discharge by leveraging accessible technology. They are less about in-person, hands-on training and more about providing timely, remote guidance, monitoring, and emotional support to help patients manage problems as they arise at home. |

**Supplementary Figure 1.** Risk of bias assessment (the incidence of stoma-related complications) of included studies

**Supplementary Figure 2.** Risk of bias assessment (patient-reported subjective outcomes) of included studies

**Supplementary Figure 3.** Forest plot of the effect of continuous care on stoma-related complications (A); sensitivity analysis (B); analysis after excluding studies with high risk of bias (C); and funnel plot for publication bias assessment (D).

**(A)**

**(B)**

**(C)**

**(D)**

**Supplementary Figure 4.** Forest plot of the effect of continuous care on QoL (A); sensitivity analysis (B); and analysis after excluding studies with high risk of bias (C).

**(A)**

**(B)**

**(C)**

**Supplementary Figure 5.** Forest plot of the effect of continuous care on self-care ability (A); sensitivity analysis (B); and analysis after excluding studies with a high risk of bias (C).

**(A)**

**(B)**

**(C)**

**(C)**

**Supplementary Figure 6.** Forest plot of the effect of continuous care on self-efficacy (A); sensitivity analysis (B); and analysis after excluding studies with a high risk of bias (C).

**(A)**

**(A)**

**(B)**

**(C)**

**Supplementary Figure 7.** Forest plot of the effect of continuous care on ostomy adjustment (A); sensitivity analysis (B); and analysis after excluding studies with a high risk of bias (C).

**(A)**

**(B)**

**(C)**

**Supplementary Figure 8.** Forest plot of the effect of continuous care on anxiety (A); and sensitivity analysis (B).

**(A)**

**(B)**

**Supplementary Figure 9.** Forest plot of the effect of continuous care on depression (A); and sensitivity analysis (B).

**(A)**

**(B)**
